# Next-generation sequencing reveals two populations of damage-induced small RNAs at endogenous DNA double-strand breaks

**DOI:** 10.1093/nar/gky1107

**Published:** 2018-11-10

**Authors:** Franziska Bonath, Judit Domingo-Prim, Marcel Tarbier, Marc R Friedländer, Neus Visa

**Affiliations:** 1 Science for Life Laboratory, Department of Molecular Biosciences, The Wenner-Gren Institute, Stockholm University, SE-106 91 Stockholm, Sweden; 2 Department of Molecular Biosciences, The Wenner-Gren Institute, Stockholm University, SE-106 91 Stockholm, Sweden

## Abstract

Recent studies suggest that transcription takes place at DNA double-strand breaks (DSBs), that transcripts at DSBs are processed by Drosha and Dicer into damage-induced small RNAs (diRNAs), and that diRNAs are required for DNA repair. However, diRNAs have been mostly detected in reporter constructs or repetitive sequences, and their existence at endogenous loci has been questioned by recent reports. Using the homing endonuclease I-PpoI, we have investigated diRNA production in genetically unperturbed human and mouse cells. I-PpoI is an ideal tool to clarify the requirements for diRNA production because it induces DSBs in different types of loci: the repetitive 28S locus, unique genes and intergenic loci. We show by extensive sequencing that the rDNA locus produces substantial levels of diRNAs, whereas unique genic and intergenic loci do not. Further characterization of diRNAs emerging from the 28S locus reveals the existence of two diRNA subtypes. Surprisingly, Drosha and its partner DGCR8 are dispensable for diRNA production and only one diRNAs subtype depends on Dicer processing. Furthermore, we provide evidence that diRNAs are incorporated into Argonaute. Our findings provide direct evidence for diRNA production at endogenous loci in mammalian cells and give insights into RNA processing at DSBs.

## INTRODUCTION

DNA double-strand breaks (DSBs) are arguably the most dangerous threats to genome stability. Once a DSB occurs, the cell initiates a DNA damage response (DDR), first trying to repair the lesion and, if this is unsuccessful, entering apoptosis (1). DSBs are repaired via two major pathways, non-homologous end joining (NHEJ) and homologous recombination (HR). Throughout the G2 and S-phases, the sister chromatid can be used as a template during HR allowing error-free repair, while in G1, cells often rely on the more error-prone NHEJ pathway, through which the two DNA ends are ligated together (1). However, the repair pathway is determined not only by the position in the cell cycle but also by the gene context, as revealed by the fact that active genes, if damaged during G1, tend to remain unrepaired for relatively long times and become repaired by HR after the replicative phase of the cell cycle (2). The repetitive ribosomal DNA (rDNA) cassette also provides exceptional circumstances for DNA repair using paralogue sequences in *cis* for HR. Therefore, damage to the rDNA can be repaired by HR independently of the position in the cell cycle (3).

Both HR and NHEJ are initiated by recognition of the DSB, which leads to the phosphorylation of histone H2AX at the break site mediated by the stress-response kinase ataxia-telangiectasia mutated (ATM) (4). Then either a 5′ to 3′ resection of the free DNA ends is initiated, which blocks NHEJ and directs repair towards the HR pathway, or the ends are protected from resection by the accumulation of NHEJ factors (1).

Research in different systems has disclosed complex relationships between DNA damage, chromatin structure and transcription (reviewed in (5)). The DDR includes signalling pathways that remodel the chromatin in the vicinity of DSBs (6,7) and shut down the transcriptional activity of nearby promoters (8,9). The DSB-induced downregulation of transcription depends on the activity of ATM, on the PBAF chromatin remodelling complex and on the distance to the DSB (9–11). In spite of this well documented transcriptional inhibition that affects promoters located near DSBs, early studies in the ascomycete *Neurospora* revealed that DNA damage triggered the production of small RNAs through a pathway that involved Dicer proteins (12). There is increasing evidence that transcription occurs at DSBs in the absence of *bona fide* promoters, and that DSB repair is governed not only by DDR proteins, but also by RNAs that are synthesized in the vicinity of the DSB. Several independent reports have shown that open DNA ends lead to initiation of transcription *in vitro* and *in vivo* and it has been suggested that this *de novo* transcripts determine the extent of DNA end resection both in yeast and mammalian cells (13–16). The discovery that small RNA biogenesis factors such as Drosha and Dicer promote the repair of DSBs in a manner that is independent of microRNAs (miRNAs) led to the proposal that short, damage-induced RNAs originate from the sequences that flank the DSB (14,17,18). These RNAs were termed diRNAs in plants (17) and DDRNAs in mammalian cells (18). The fact that a fraction of Dicer is phosphorylated upon DNA damage and recruited to DSBs gives further support to this proposal (19,20).

Several indirect lines of evidence suggest that diRNAs are important for resolving DSBs. For example, the RNA binding capacity and catalytic activity of Argonaute-2 (AGO2) are required for the recruitment of Rad51 to DSBs (21), and diRNA mimics are able to rescue the effects of Drosha and Dicer depletions on the recruitment of chromatin remodelling factors to DSBs (22). Transcription at DSBs has been linked to the DDR and it has been proposed that each DSB produces a unique set of site-specific RNAs that are necessary for DDR activation (16). Furthermore, next-generation sequencing confirmed the production of diRNAs in plants at a transgenic reporter locus harbouring a cleavage site for an endonuclease (17). DiRNAs were also detected after Cas9-mediated cleavage in unique genomic locations in the *Drosophila* genome (23) and at DSBs within telomeres of mammalian cells (24). However, attempts to sequence diRNAs in mammalian cells at unique genomic loci in a natural chromatin context have been unsuccessful and their existence in genetically unperturbed cells has been questioned (14). Moreover, the functional importance of diRNAs has been challenged by the observation that the DNA repair defects caused by Dicer depletion can be rescued by a miRNA mimic, which suggests that the contribution of Dicer to DSB repair is indirect (25).

Four mutually exclusive hypotheses regarding diRNA biogenesis have been proposed. One, diRNAs are made independently of the location and sequence of the DSBs and serve as guides for the recruitment of DDR factors through a mechanism that involves RNA-RNA pairing (16,18,24). If this is the case, difficulties in revealing diRNAs at unique DSBs in mammalian cells could be due to their low abundance or fast turnover. Two, the extent of diRNA formation depends on local transcription and therefore only genic regions will give rise to diRNAs (13,23,26). In this scenario, diRNA production could contribute to the particular DNA repair features of transcribed genes (27) or to the degradation of aberrant mRNAs produced at the damaged gene locus, as shown in *Drosophila melanogaster* (23). Three, diRNAs are a special response to DSBs produced in repetitive regions. In support of this proposal, studies in the ascomycete *Neurospora* showed that the production of small damage-induced RNAs is dependent on the repetitive nature of the damaged DNA (12,28). A small RNA response has also been recently reported at unprotected telomeres (24). And four, diRNAs do not exist *in vivo* and previously detected diRNAs were peculiarities of specific reporter systems (14,29).

Whether diRNAs exist and whether their production depends on certain conditions, such as the transcriptional status or the repetitive nature of the locus that harbours the DSB, are important questions in order to understand the role of RNA in DSB repair. Here, we investigate these questions using the homing endonuclease I-PpoI in mouse and human cell lines. The I-PpoI system offers the advantage of introducing DSBs in repetitive 28S ribosomal DNA (rDNA) loci as well as in unique genic and intergenic loci, in genetically unperturbed cells. We find that exhaustive next-generation sequencing does not provide evidence of diRNA production in unique genic or intergenic sequences. However, DSBs in the rDNA robustly induce the production of diRNAs and we show the existence of two types of diRNAs with characteristic length and terminal nucleotide signatures. Surprisingly, our analyses of diRNAs in mouse knock-out (KO) cell lines show that Drosha and its binding partner DGCR8 are dispensable for diRNA production and that only one of the two diRNA subpopulations requires Dicer processing. Last, we present evidence for the incorporation of rDNA-derived diRNAs into Argonaute effector complexes, suggesting that they may play functional roles.

## MATERIALS AND METHODS

### Cell culture

HeLa and DIvA cells (30) were cultured at 37°C in 5% CO_2_ in Dulbecco’s modified Eagle’s medium (DMEM) medium (Sigma, D629) supplemented with 10% fetal bovine serum (FBS) and 1% penicillin/streptomycin. The DIvA cells were maintained in culture medium containing 1 μg/ml puromycin. The mESC lines used have been previously described: mESC drosha KO and the corresponding parental line (31), mESC dicer KO and the corresponding parental line (32) and mESC *Dgcr8* KO (33). The parental line of the drosha KO was used as control mESC line unless otherwise stated. mESCs were cultured in LIF+serum medium composed of DMEM (Gibco, 41965-039) supplemented with 0.01% mLIF, 15% FBS, 2% penicillin/streptomycin, 1% glutamine, 1% sodium pyruvate, 1% minimum essential medium—non-essential amino acids (MEM NEAA), and 0.2% beta-mercaptoethanol at 37°C in 5% CO_2_ in gelatin-coated flasks.

### SiRNA transfection

The day before transfection, HeLa cells were seeded in 6-well plates with 50–60% confluence in 2 ml DMEM supplemented with 10% FBS and 1% penicillin/streptomycin. SiRNAs were mixed with 4 μl of Lipofectamine RNAiMAX (Invitrogen) in 1 ml Opti-MEM (Gibco) per well and added to the plates, and the cultures were then incubated for 48 h. The siRNA for Drosha and the corresponding control were Silencer siRNA from ThermoFisher (CatNo: AM16708, ID:126719 and CatNo: 4390844, respectively). They were used at a final concentration of 25 nM.

### Plasmid transfection and transcription inhibitor treatments

HeLa cells were seeded at 70–80% confluence in 6-well plates in 2 ml DMEM supplemented with 10% FBS without penicillin/streptomycin the day before transfection. In total, 2.5 μg of pOPRSVI-I-PpoI plasmid was transfected with Lipofectamine 3000 (Invitrogen) as described in the manufacturer’s protocol. In some experiments, transcription inhibitors were added 12 h prior to harvesting at the following concentrations: α-amanitin 5 μg/ml (Sigma, A2263), DRB 150 μM (Sigma, D1916), actinomycin D 0.05 μg/ml (Sigma, A9415). CX-5461 (S2684, Selleckchem) was used at 100 nM for 20 h.

3 × 10^5^ mESCs were seeded in 6-well plates in 3 ml LIF+serum medium the day before transfection. A total of 2.5 μg of pOPRSVI-I-PpoI plasmid was transfected with Lipofectamine 3000, again as described by the manufacturers protocol. For small RNA sequencing, the cells were harvested 36 h after transfection. Cordycepin (Sigma, C3394) was used at a final concentration of 500 μM and added to the cultures 24 h after transfection.

### RT-qPCR

RNA (1 μg) was treated with 1 unit DNase I (Thermo Fisher) for 60 min and reverse-transcribed using random primers (Thermo Fisher Scientific) and SuperScript II or III (Invitrogen). The resulting cDNA was quantified by quantitative polymerase chain reaction (qPCR) in a RotorGene (Qiagen) or a StepOnePlus (Thermo Fisher Scientific) using Power SYBR Green PCR Master Mix (Kapa Biosystems). The following primers were used: Gapdh_int_fw GCAATGAGTGAGGTCCTGCAC, Gapdh_int_re TATGTAGGCAGTGGGGAGACAG, Gapdh_ex_fw CCTGCTTCACCACCTTCTTGATGTC, Gapdh_ex_re CAAGGTCATCCCAGAGCTGAACG, 45S_fw CTCCGTTATGGTAGCGCTGC, 45S_re GCGGAACCCTCGCTTCTC, ARPP_f GCACTGGAAGTCCAACTACT, ARPP_r TGAGGTCCTCCTTGGTGAACAC.

### Strand specific RT-qPCR (ss-RT-qPCR)

RNA was purified and DNase-treated as above. For each reverse transcription reaction, strand-specific primers from the region of interest and the control gene were used. RNA levels in untreated samples and in samples treated with actinomycin D were normalized to the levels of ARPP or GAPDH (exon primers as above). In samples treated with RNAPII inhibitors, the RNA levels were normalized to the 28S rRNA (reverse, upstream). The resulting cDNA was quantified by qPCR in a RotorGene (Qiagen) using Power SYBR Green PCR Master Mix (Kapa Biosystems). Primer sequences for ss-RT-qPCR reactions were as follows (in 5′ to 3′): Ryr2_500up_f ATTCTGGGCAGAGGATGAGA, Ryr2_500up_r TCTGGAAGGCATCCTTTGTT, Ryr2_500dw_f TCAAGACCCATCAGTGCTGT, Ryr2_500dw_r AATCCCTGCCTTTGTTTTCC, 28S_900up_f GGGGGAGAGGGTGTAAATCT, 28S_900up_r TTGCCGACTTCCCTTACCTA, 28S_600dw_f CGATGTCGGCTCTTCCTATC, 28S_600dw_r AACCTGTCTCACGACGGTCT, ARPP_f GCACTGGAAGTCCAACTACT, ARPP_r TGAGGTCCTCCTTGGTGAACAC.

### Determination of I-PpoI cleavage efficiency

HeLa cells were transfected with the pOPRSVI-I-PpoI plasmid or mock transfected. The cells were resuspended in lysis buffer (1 × TE, 0.05% sodium dodecyl sulphate (SDS)) containing 0.05 mg/ml Proteinase K at 60°C for 4 h. Genomic DNA was extracted with phenol-chloroform-isoamylalcohol following standard procedures. A total of 100 ng of gDNA was used in a qPCR using primers upstream and downstream of the DSB. The following primers were used: AAMDC_DSB_fw GGCCAGTATGAATGATCATCAGG, AAMDC_DSB_re GCGTCACTAATTAGATGACAAGGC, chr7_68527501_fw TGCTCTATGAAAACACCATTAAG, chr7_68527501_re GCGCGTCACTAATTAGATGAC, Dab1_DSB_fw CCATACGTGGCAGAGTGTG, Dab1_DSB_re GCCTCACTGAAGACTTGGTG, SLCO5A1_DSB_fw CTCGTTCATCCATTCATGCGC, SLCO5A1_DSB_re CTGTGTCTCATGGGTAGCTTAATC, Ryr2_R1_f GAGGTGGGCAAGAATGAGAA, Ryr2_500dw_r AATCCCTGCCTTTGTTTTCC, 28S_100up_f CAGGGGAATCCGACTGTTTA, 28S_100up_r GGGGCCTCCCACTTATTCTA.

### Argonaute RNA immunoprecipitation

HeLa cells were seeded the day before transfection and grown to 80 % confluence in 6-well plates. The cells were transfected with 2.5 μg pOPRSVI-I-PpoI plasmid per well using the standard protocol for Lipofectamine 3000. The cells were scraped off 36 h after transfection and RNA immunoprecipitation was performed using the Magna RIP™ RNA-Binding Protein Immunoprecipitation Kit (Merck) following the manufacturers instructions and using 6 μg of anti-AGO antibody from Abcam (ab57113, antibody A) or Diagenode (Cat. No. C15200167, antibody B). These antibodies recognize the human Argonaute 1–4 proteins (34). The recovered RNA from RIP and 1 μg of input RNA were used for standard small RNA sequencing library preparation using the Illumina TruSeq small RNA library preparation kit (Illumina, December 2014) and sequenced on an Illumina NextSeq 500 sequencing machine for 75 cycles.

### Small RNA library preparation and sequencing

Total RNA was extracted from HeLa or mESC cells using TRIZOL, and libraries were prepared following the instructions of the TruSeq small RNA library preparation protocol (Illumina, December 2014). Small RNA spike-ins (Exiqon, product no. 800100) were dissolved in 50 μl of nuclease-free water for the analysis of RNAs from Drosha RNAi, *Dgcr8* KO, dicer KO and cordycepin treatments; and in 150 μl for the drosha KO experiments. In all cases, 1 μl of the spike-in solution was added to 1 μg of total RNA input before library preparation. The cDNA was size fractionated in gel and inserts derived from 20 to 33 nt small RNAs were sequenced. The barcoded cDNA libraries were pooled and run on an Illumina NextSeq500 sequencer for 75 cycles. The libraries had an average depth of ∼10 million reads.

### Small RNA-seq pre-processing

A custom Perl script was applied to pre-process de-multiplexed sequencing reads (available upon request). Reads were trimmed to 50 nt, and those containing more than 50% of low-quality base assignments (having a Phred quality score <20) were excluded. Adapters were trimmed by searching for matches to the first 8 nt of the adapter in the 3′ ends of the reads. If the 8-mer was found, the last match and the subsequent nucleotides were trimmed. Then reads containing ambiguous or highly repetitive nucleotides (e.g. N), or sequences shorter than 18 nt were discarded. Identical reads were collapsed to a single read carrying the read count in the identifier.

### Small RNA-seq analyses of the 28S locus

We curated a ribosomal RNA library based on RNA sequences retrieved from NCBI (www.ncbi.nlm.nih.gov), Silva (arb-silva.de) and the Ensembl database (www.ensembl.org). Reads were subsequently mapped against our rRNA library using Bowtie v1.12 (http://bowtie-bio.sourceforge.net) with options ‘-v 0 -q -k 1 -7 - -best’. Small RNA abundance at the 28S rDNA locus was either normalized to total reads mapping to the 28S rDNA (coverage blot in the HeLa time course), to total collapsed reads or to spike-in read counts, as described in the main text. In order to retrieve the abundance of spike-ins, the adapters were removed from the original fastq files using cutadapt v1.11 (https://github.com/marcelm/cutadapt) and the trimmed reads were mapped to spike-in sequences using Bowtie with the options ‘-v 0 -q -k 1 - -best’. To visualize single read alignments that mapped to the antisense strand of the 28S rDNA close to the I-PpoI site, the Integrative Genomics Viewer v2.4.2 (http://software.broadinstitute.org/software/igv) was used. Coverage plots, read length distributions and terminal nucleotide analyses were performed using the R software package v3.2.2 (www.r-project.org) as follows. For coverage plots, the distance to the break site was calculated separately for reads mapped to the sense and antisense strands, and the nucleotide density at each position was determined. Coverages of replicates were averaged and the means of 20 nt bins were plotted relative to the I-PpoI site. The read length distribution was generated on collapsed reads for reads that mapped to the 28S rDNA upstream of the DSB in antisense direction. To analyse the nucleotide biases, collapsed reads that mapped to the 28S rDNA locus upstream of the DSB in antisense direction were extracted and separated into two fractions, one with reads of length 22 nt, and one with reads of 25 nt or longer. The first and last nucleotides of reverse-complemented reads were obtained, and the proportion of each nucleotide calculated.

### Small RNA-seq analysis at unique genomic loci

For analysis of diRNAs at unique genomic loci, the genome assembly for either human (hg19) or mouse (mm10) was retrieved from genome.ucsc.edu. I-PpoI sites were identified genome-wide by mapping the I-PpoI consensus sequence (5′-CTCTCTTAAGGTAGC-3′) to the human and mouse genomes using bowtie with the options ‘-v 0 –a’. After pre-processing, reads that mapped to tRNAs, ribosomal RNAs or miRNAs allowing one mismatch were discarded and remaining reads mapped to the genomes using bowtie with the options ‘-v 0 - -best -k 1’. Reads mapping to a region of 5 kb upstream or downstream of the identified I-PpoI sites were extracted using a custom Bash script. Using R, reads mapping to multiple genomic loci were discarded and remaining reads were divided into forward strand, reverse strand, genic and intergenic regions based on manual inspection of the respective I-PpoI sites. The sum of reads mapping to the regions antisense, upstream and sense, downstream of the I-PpoI sites were visualized using R. A full list of libraries used for the analysis is shown in Supplementary Table S1.

### Small RNA-seq analyses of miRNA expression

MiRNA precursor sequences were retrieved from miRBase version 20 (www.mirbase.org). The profiles of mature miRNA expression across the different conditions were determined using the quantifier module of the miRDeep2 package (https://github.com/rajewsky-lab/mirdeep2) allowing no mismatches. Reads assigned to identical mature miRNAs were summed. Reads for each sample were subsequently normalized to spike-ins per million and replicates were averaged. For the 200 mature miRNAs with the highest expressions in the control, we determined the fold change between the miRNA levels in the KO and the control, and plotted these against their normalized expression levels in the control samples.

### rDNA copy number determination

Genomic DNA was purified from HeLa cells and the number of rDNA copies was determined by qPCR relative to ARPP. The qPCR reactions were carried out in a RotorGene using Power SYBR Green PCR Master Mix. The primers used were the ARPP and 28S_900up specified above. The results from three independent quantifications were averaged and the average was used for the calculations presented in the Supplementary Table S4.

### Enrichment analysis in AGO-RIPseq

The sequences of snoRNAs and tRNAs (hg19) were downloaded from NCBI. Reads not mapping to miRNAs or the rDNA locus were mapped to the snoRNA or tRNA library with bowtie v0.1.2 and the options ‘-k 1 -v 0 - -best’. Collapsed diRNA reads (mapping to the reverse strand of the 28S rDNA locus), collapsed snoRNA reads, collapsed reads mapping to the 28S rDNA forward strand, collapsed tRNAs and uncollapsed miRNA reads were normalized to total reads passing quality control of each sample. Subsequently, for each replicate, the fold change between input and IP was calculated. The fold changes were normalized to the fold change of the median miRNA. The normalized fold changes from RIP replicates were averaged.

### Estimation of diRNA copies per cell

Collapsed reads mapping to the 28S region in antisense direction and unique genomic loci were normalized to the expression of miR-15a in the same samples using the formula:

\begin{equation*}\frac{{{\rm{miR}} \hbox{-} 15{\rm{a}}\ \left( {{\rm copies}\;{\rm per}\;{\rm cell}} \right)}}{{{\rm{miR}} \hbox{-} 15{\rm{a}}\left( {{\rm reads}} \right)}}\times \it{diRNA} \left( {{\rm reads}} \right)\end{equation*}

The number copies per cells for this miRNA was experimentally determined previously (35). The small RNA libraries used are listed in Supplementary Table S1 (HeLa samples, I-PpoI transfected only).

### Immunofluorescence

mESCs were transfected with either 2.5 μg pEGFP-C3 plasmid (Clontech) or a mixture consisting of 1 μg pEGFP-C3 and 1.5 μg pOPRSVI-I-PpoI. The cells were fixed with 4% formaldehyde 24 h after transfection, permeabilized with 0.1% Triton X-100 for 15 min and processed for immunofluorescence following a standard protocol. The primary antibody was against phosphorylated H2AX (#9718, Cell Signaling Technology). The secondary antibody was a goat anti-rabbit Alexa Fluor 633 (A-21070, ThermoFisher Scientific). Coverslips were mounted using VectaShield with DAPI (VectorLabs) and the slides were visualized and imaged in a LSM780 confocal microscope (Carl Zeiss) with an optical thickness of 0.9 μm.

### Statistical analysis

To analyse the enrichment for reads of length 21–22 nt in the diRNA population, reads of 21 or 22 nt that mapped to the antisense 28S rDNA locus were considered successes and all reads from the same locus between 20 and 23 nt were considered trials, for a binomial test of whether the number of successes was greater than the number expected from random sampling. The command used for the binomial test in the R software package was as follows: binom.test(‘reads with 21 or 22 nt’, ‘reads with 20 to 23 nt’, *P* = 0.5, alternative = ‘greater’). Statistical testing for significance in ss-RT-qPCRs was performed as a one-tailed *t*-test.

### Protein detection

Relative levels of protein expression were determined by western blotting. Cells were lysed in 2× Laemmli buffer and separated on an 8% SDS-polyacrylamide gel. Proteins were transferred to a polyvinylidene difluoride (PVDF) membrane using a standard wet transfer procedure. The following antibodies were used for detection: primary antibodies against Drosha (Thermo Fisher, MA5-14784), Tubulin (Sigma, T5168), Argonaute (Diagenode, C15200167) and horseradish peroxidase (HRP)-conjugated secondary antibodies against mouse (Sigma, SAB3701066) or rabbit (DAKO, P0448) immunoglobulins. The ECL blotting reagent was obtained from Sigma (GERPN2109) and the reaction was monitored on a Biorad ChemiDoc XRS+ using Image Lab. Quantification of protein bands was done with Fiji version 2.0.

## RESULTS

### DSBs in natural repetitive loci give rise to diRNAs in mammalian cells

**Figure 1.**
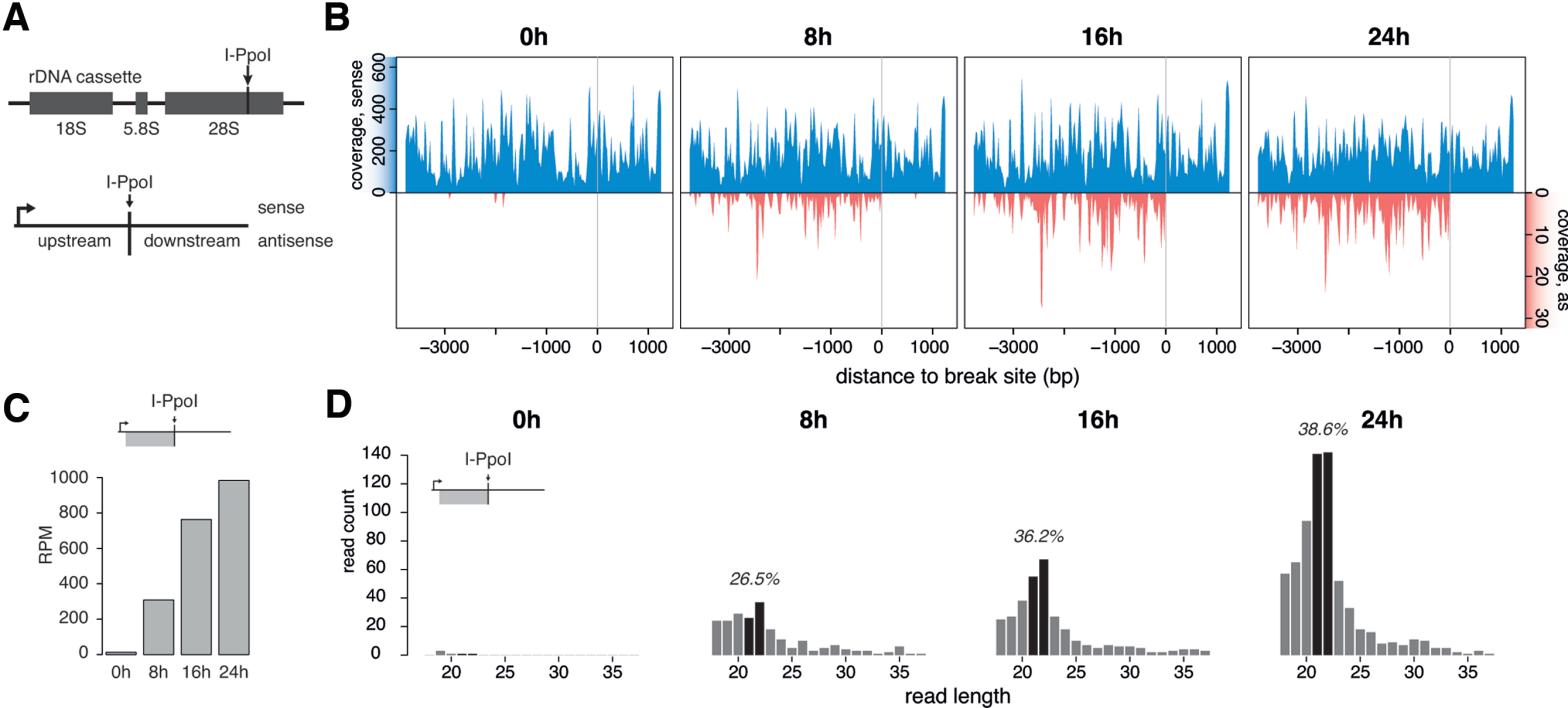
DiRNAs originate from endogenous DSBs in human cells. (**A**) Schematic representation of the 28S rDNA (upper part) and definition of areas around DSBs (lower part). The arrow indicates the location of the I-PpoI target site. The lower panel defines the position and polarity of the transcripts analysed. Upstream and downstream are relative to the I-PpoI cleavage site. Sense and antisense are according to the canonical transcription start of the rDNA locus (arrow). Sense transcripts have the same polarity as the 28S rRNA. (**B**) Small RNA reads from HeLa cells transfected with a plasmid that expressed I-PpoI analysed at different times after transfection, as indicated. The reads were mapped to the 28S rDNA locus and normalized to total collapsed 28S rRNA read counts. The grey vertical line in each plot indicates the position of the I-PpoI cleavage site in the 28S locus. The top (blue) and bottom (red) parts of the plot show the coverage in the sense and antisense strands, respectively. The reads in the sense strand (blue) are assumed to be predominantly degradation products of rRNA. (**C**) Quantification of diRNA reads in the 28S rDNA locus expressed as reads per million (RPM) of total collapsed reads. The plot includes only upstream, antisense reads. (**D**) Length distributions of antisense small RNAs derived from the 28S rDNA locus, upstream region, at different times after transfection with the I-PpoI expression plasmid. The black bars highlight reads of 21 and 22 nt. The percentage of the 21–22 nt population is displayed. Enrichment of the 21–22 nt fraction is significant at 16 h (*P* = 1.85 × 10^−5^) and 24 h (*P* = 1.77 × 10^−11^) in an exact binomial statistical test.

In order to investigate the production of small RNAs at DSBs we utilized the homing endonuclease I-PpoI, which has a well-characterized recognition motif in the 28S ribosomal DNA locus (Figure 1A) and additional recognition motifs in unique genomic loci (see Supplementary Tables S2 and 3). We transfected HeLa cells with a plasmid for I-PpoI expression and we sequenced the small RNA fractions of cells harvested at different time points after transfection. The levels of I-PpoI expression and the cleavage efficiency were monitored by RT-qPCR and qPCR, respectively (Supplementary Figure S1A). Transcription at the rDNA locus was not affected by the expression of I-PpoI as the relative 45S rRNA levels were unchanged after transfection (fold change I-PpoI-transfected compared to mock transfected cells 1.025 ± 0.293, *n* = 3 independent experiments).

From here on, the region located between the DSB and the promoter of the gene that hosts the DSB will be referred to as ‘upstream’, and the region between the DSB and the end of the host gene as ‘downstream’. ‘Sense’ and ‘antisense’ are relative to the transcriptional start site of the host gene (Figure 1A).

In mock transfected cells (control cells that did not express I-PpoI), small RNAs that aligned to the sense strand of the 28S locus were present (blue signals in Figure 1B). The vast majority of these sense RNAs were degradation intermediates of the rRNA. However, transcripts that aligned to the antisense strand were virtually undetectable (Figure 1B, 0 h). Eight hours after I-PpoI transfection, small RNAs from the antisense strand started to accumulate and continued to be present until the end of the time course, 5 days after transfection (red signals in Figure 1B and C; Supplementary Figure S1B and C). Interestingly, the antisense reads mapped exclusively upstream to the I-PpoI cleavage site, and extended throughout the 28S rDNA and into the 5S and 18S regions. As the small antisense RNAs induced upstream of I-PpoI DSBs were DNA-damage dependent, we refer to them as ‘diRNAs’.

We could detect diRNA formation upstream of the I-PpoI cleavage site, but not downstream. Transcriptional events starting at DSBs should proceed in both directions. RNA polymerases can transcribe only in the 5′ to 3′ direction and, accordingly, transcription initiated at the DSB will proceed upstream of the break in the antisense direction, and downstream of the break in the sense direction. Therefore, the sequence of the downstream sense RNAs will be identical to that of the rRNA and as such we cannot rule out bidirectional transcription because our analysis cannot distinguish between I-PpoI-induced transcripts and transcripts from background transcription in the sense strand.

A closer look at the diRNAs closest to the DSB revealed that their 5′ ends mapped between 8 and 16 nt away from the I-PpoI cleavage site. Reads as close as 2 nt away from the break occurred at later time points (four and five days after transfection, Supplementary Figure S2). The lack of reads that reach the cleavage site could be due to the requirement of the RNAPII initiation complex for a minimum sequence to bind to, upstream of the first transcribed nucleotide (13,36). However, experiments analysing small RNAs production in cell free extracts did show that polymerases do have the potential to transcribe even the very first nucleotide of free DNA ends of linearized plasmids (16).

The diRNAs reported in previous studies had a length between 21–24 nt, typical for Dicer products (17). When we analysed the diRNAs produced in the time series reported above, we found that at 8 h after transfection they were predominantly shorter than 25 nt. As time proceeded, diRNAs of lengths 21–22 nt became the dominant fraction, forming a clear peak as early as 16 h after transfection (Figure 1C and D; Supplementary Figure S1C). However, diRNAs shorter than 21 nt and longer than 22 nt were consistently detected and comprised ∼60-70% of the total diRNA population. It is important to point out that the upper and lower read length cutoffs in our small RNA-seq analysis arose for technical reasons, and we cannot exclude the existence of shorter or longer transcripts.

**Figure 2.**
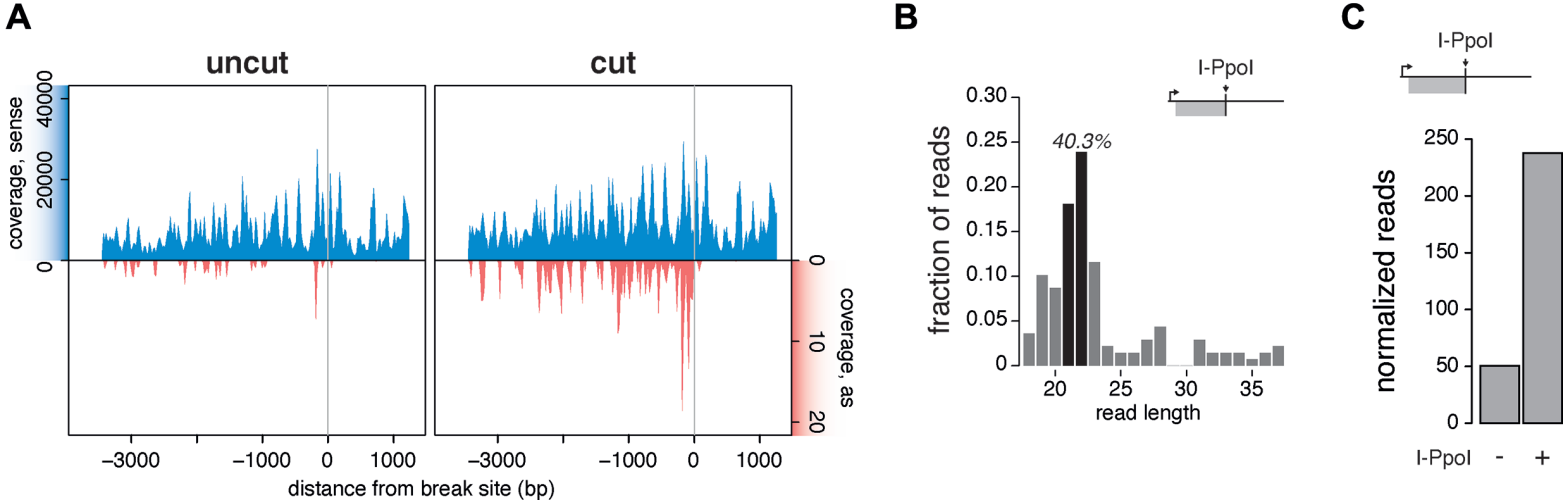
DiRNAs originate from endogenous DSBs in murine cells. (**A**) Small RNA reads from mESC cells transfected with a plasmid that expressed I-PpoI analysed 36 h after transfection. The reads were mapped to the 28S rDNA locus and normalized to spike-ins. The grey vertical lines indicate the position of the I-PpoI cleavage site in the 28S locus. The top (blue) and bottom (red) part of the plot show the coverage in the sense and antisense strands, respectively. (**B**) Length distributions of antisense small RNAs derived from the 28S rDNA locus, upstream region, at 36 h after transfection with the I-PpoI expression plasmid. The black bars highlight reads of 21 and 22 nt. The percentage of the 21–22 nt population is displayed. (**C**) Quantification of diRNA reads in the 28S rDNA locus expressed as collapsed reads per million spike-ins. The plot includes only upstream, antisense reads.

To establish whether the production of diRNAs was general to mammalian cells, we transfected mouse embryonic stem cells (mESCs) with I-PpoI and sequenced the small RNA fractions. DNA cleavage by I-PpoI in mESCs was assessed by immunofluorescent staining of I-PpoI transfected cells with an antibody against phosphorylated H2AX (Supplementary Figure S3A). As in HeLa cells, the levels of small RNA fragments in the sense direction were high in untransfected cells. Importantly, I-PpoI cleavage caused a clear increase in diRNAs upstream of the break in the antisense direction, and these diRNAs had a predominant length of 21–22 nt (Figure 2 A and B).

The diRNAs that we detected after DSB induction could stem from an already existing transcript, which is degraded or processed upon DSB formation, or from *de novo* transcription. To distinguish between these two possibilities, we treated mESCs with the general RNA polymerase inhibitor cordycepin after transfection with I-PpoI and we sequenced the small RNA fraction. Even though cordycepin treatment increased background levels of small RNAs even in uncut controls, the treatment reduced the formation of diRNAs considerably (Supplementary Figure S4) confirming that diRNAs at repetitive loci are products of a *de novo* transcription event.

### DiRNAs are not detected at unique genic or intergenic loci

An advantage of the I-PpoI system is that it produces DSBs not only in the highly expressed and repetitive rDNA locus, but also at unique genomic loci. We confirmed by Sanger sequencing that unique I-PpoI target sequences were intact in the HeLa cells used for this study (Supplementary Figure S5).

**Figure 3.**
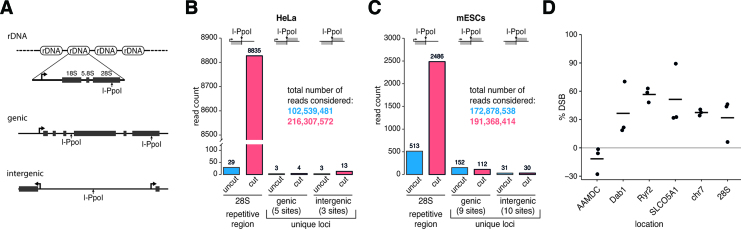
DiRNAs arise from repetitive, but not from non-repetitive genomic loci in mammalian cells. (**A**) Schematic definition of repetitive ribosomal (rDNA), genic and intergenic regions used in (B and C). I-PpoI sites in genic regions can be located in exons or introns. I-PpoI sites defined as intergenic do not overlap with any annotated gene. (**B** and **C**) Total read counts of collapsed reads mapped to the 28S rDNA locus or to genic and intergenic loci in HeLa cells (B) or mESCs (C). The genome coordinates of the loci included in the analysis are listed in Supplementary Tables S2 and S3. For an overview of underlying samples see Supplementary Table S1. Read counts per locus are listed in Supplementary Table S4. (**D**) The efficiency of I-PpoI cleavage at selected loci was quantified by qPCR using primers spanning the I-PpoI motif on genomic DNA and expressed as percentage of DSBs. The analysis was carried out 24 h (for Ryr2, Dab1 and SLCO5A1) or 36 h (for AAMDC, chr7 and 28S rDNA) after transfection. The ARPP locus is not cleaved by I-PpoI and was used for normalization.

To get the best possible resolution of diRNAs at these unique loci, we pooled sequence data from numerous sequencing experiments, comprising altogether 700 million reads distributed between mock and I-PpoI transfected samples (Supplementary Table S1). We classified the unique sites into genic and intergenic to see whether transcription prior to DSB induction was required for diRNA production (Figure 3A). In concordance with previous reports (14,29), we could not detect diRNA at unique endogenous DSBs in either human (Figure 3B and Supplementary Table S4) or mouse (Figure 3C and Supplementary Table S5) samples in spite of the great sequencing depth (HeLa: 216 million reads, mESCs: 191 million reads, considering only cut samples) and the high DSB frequencies (Figure 3D). A few sequencing reads were detected at the unique genomic loci, but these reads were observed at similar levels in both mock and I-PpoI transfected cells (Supplementary Tables S4 and 5). Moreover, these reads did not show a preferred read length of 21–22 nt (Supplementary Figure S6).

We estimated that the average abundance of diRNAs from the 28S locus in HeLa cells transfected with the I-PpoI plasmid was ∼28 molecules per cell based on comparison to the known expression levels of miR-15a (see ‘Materials and Methods’ section) (35,37). We also quantified by qPCR the rDNA copy number in the HeLa cells that were used for this study and estimated that the average number of diRNA molecules per cleaved I-PpoI site in the 28S locus in the haploid genome was 1.12. The number of small RNAs originating from unique loci was approximately two orders of magnitude lower (Supplementary Tables S4).

### *De novo* transcription at DSBs is not sufficient for diRNA production

**Figure 4.**
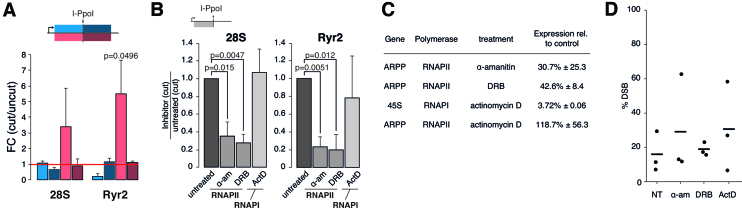
RNAPII transcribes sequences adjacent to DSBs in repetitive and non-repetitive genomic sites. (**A**) Quantitative RNA analysis in regions flanking I-PpoI cleavage sites in the 28S and *Ryr2* loci. HeLa cells were transfected with the I-PpoI plasmid or mock transfected. RNA levels were quantified by ss-RT-qPCR using four PCR primer-pairs designed to amplify independently RNAs from the upstream and downstream regions in each of the two directions (sense or antisense), as depicted in the figure. The plot shows the fold change between the RNA levels in cells that express I-PpoI compared to control, mock transfected cells. The bars show averages from three independent experiments. The error bars represent standard deviations. The *P*-values result from a one-tailed Student’s *t*-test. (**B**) Ss-RT-qPCR comparing RNA levels at the 28S and *Ryr2* loci after I-PpoI transfection in control HeLa cells or cells treated with different RNA polymerase inhibitors 12 h after I-PpoI transfection. The error bars display standard deviation from three independent experiments. The *P*-values result from a two-tailed Student’s *t*-tests. (**C**) The levels of ARPP mRNA and 45S rRNA were quantified by RT-qPCR in control cells and in cells treated with different inhibitors to inhibit the action of the indicated RNA polymerase. All experiments were performed in three independent experiments and the standard deviations are given. (**D**) I-PpoI cleavage at the *Ryr2* locus was quantified in non-treated control cells (NT) and in cells treated with different transcriptional inhibitors. The percentage of DSBs was quantified by qPCR on genomic DNA with primers spanning the I-PpoI recognition motif and was normalized to ARPP, which is not cleaved by I-PpoI.

It has been reported that diRNAs are processed from longer damage-induced long non-coding RNAs (dilncRNAs), which are transcribed by RNAPII (16), and we were interested in determining whether dilncRNA production was also restricted to repetitive sequences. We analysed the synthesis of dilncRNAs at two I-PpoI sites: the I-PpoI target site at the 28S rDNA locus, and a previously described I-PpoI target site within the *Ryr2* protein-coding gene (38). The *Ryr2* pre-mRNA is ∼0.8 Mb long and contains 104 introns. Strand-specific reverse transcription quantitative PCR (ss-RT-qPCR) showed that I-PpoI caused a consistent upregulation of antisense transcripts upstream of the break in both the 28S rDNA and *Ryr2* loci, whilst the sense strand of the same samples upstream of the break was not upregulated (compare the bright red and blue bars in Figure 4A).

The ribosomal locus can be transcribed in the antisense direction by RNAPII in response to stress. This stress-induced transcript is a long non-coding RNA that is transcribed from a promoter in the intergenic rDNA spacer and extends into the 28S region (39,40). We performed ss-RT-qPCR for the antisense strand downstream of the break in order to determine whether the observed increase in antisense RNA levels was due to a transcript originating at the DSB, and not the result of a longer transcript triggered by a stress response. RNA levels downstream of the I-PpoI cleavage site did not increase (dark red bars in Figure 4A). Thus, we conclude that the increase in antisense RNA levels in the upstream region was due to transcripts that were initiated at or near the break, not to the induction of the stress-induced nucleolar transcript. To further rule out that nucleolar stress linked to the DDR was the cause of the observed increase in upstream RNA levels, we showed that DSBs produced in other genomic sites, using a different restriction enzyme, did not induce dilncRNA production in the 28S locus (Supplementary Figure S7).

We then determined which RNA polymerase (RNAP) is responsible for the transcription at DSBs. α-Amanitin and DRB inhibit RNAPII. DRB inhibits transcriptional elongation by RNAPII by blocking carboxyl-terminal domain kinases, while α-amanitin acts by binding directly to the active site of the polymerase (41). Treatment of cells with either drug before DSB induction significantly reduced the antisense transcription upstream of the I-PpoI site in the 28S rDNA and *Ryr2* loci after I-PpoI transfection (Figure 4B). This did not occur when transcription by RNAPI was inhibited by a low concentration of actinomycin D (Figure 4B) (41). The effect of the transcription inhibitors on the expression of control genes was verified by RT-qPCR (Figure 4C). The I-PpoI cleavage efficiency was not affected by the inhibitor treatments, as shown by a qPCR analysis of DSB frequency at the *Ryr2* locus (Figure 4D).

We also carried out experiments of transcription inhibition using a more specific RNAPI inhibitor, CX-5461 (42). HeLa cells were transfected with the I-PpoI plasmid, treated with CX-5461 for 20 h and analysed by ss-RT-qPCR. We detected increased levels of upstream antisense dilncRNAs at the 28S locus (average fold change 2.31 ± 1.04 compared to mock transfection, *n* = 3 independent experiments), which confirms that dilncRNAs are not RNAPI products. CX-5461 inhibits RNAPI transcription initiation and we speculate that in the absence of sense transcription, the rDNA is more readily transcribed by RNAPII in the antisense direction.

The fact that the synthesis of antisense RNAs in the upstream region can be detected at the *Ryr2* locus (a RNAPII transcribed gene) as well as at the 28S locus (which is transcribed by RNAPI) suggests that dilncRNAs are produced by RNAPII irrespectively of which polymerase canonically transcribes the locus. We also conclude that transcription at DSBs is a common feature of DSBs in repetitive as well as non-repetitive regions. Furthermore, our results suggest that dilncRNAs are required but not sufficient for diRNA production.

### Drosha and DGCR8 are not required for diRNA biogenesis

**Figure 5.**
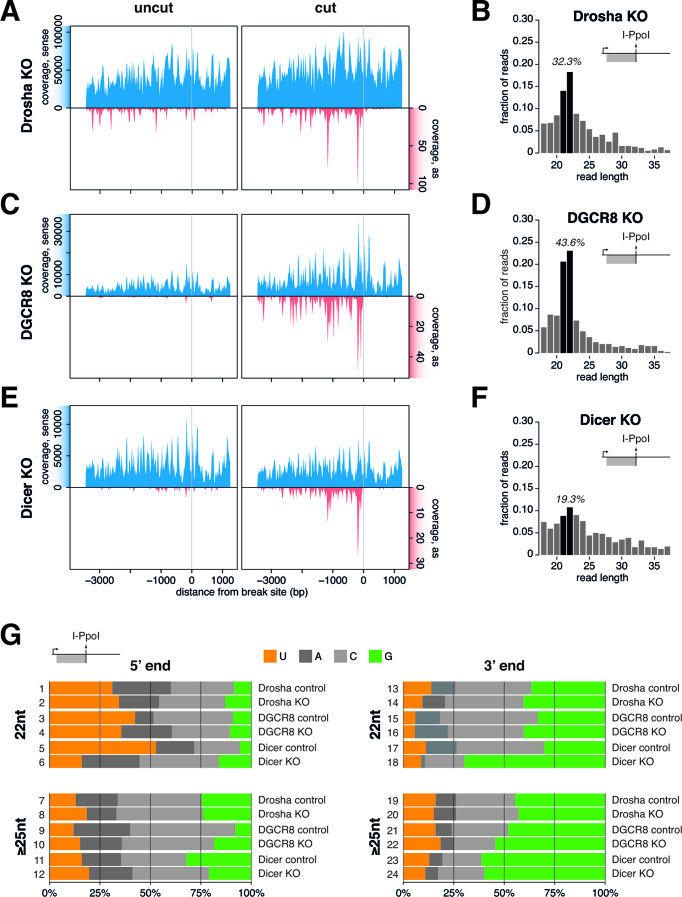
Dicer, but not Drosha or DGCR8, is required for the biogenesis of a subset of diRNAs. (**A, C** and **E**) Coverage of collapsed small RNA reads mapping to the 28S rDNA locus normalized to spike-ins per million in mESC KO lines for drosha (A), *Dgcr8* (C) or dicer (E). In all cases, mock transfected cells (uncut) and cells transfected with I-PpoI (cut) were analysed in parallel 36 h after transfection. The reads from two (dicer, *Dgcr8*) or three (drosha) biological replicates were averaged. The grey vertical lines indicate the I-PpoI cleavage site. (**B, D** and **F**) Length distributions of diRNAs produced at the 28S rDNA locus (upstream, antisense) in I-PpoI transfected (cut) drosha, *Dgcr8* and dicer KO mESC lines, as indicated. The percentage of the 21–22 nt population is displayed. Enrichment of the 21–22 nt fraction was significant for drosha KO (*P* = 6.35 × 10^−9^) and *Dgcr8* KO (*P* = 1.06 × 10^−15^), but not for dicer KO (*P* = 0.085) mESCs in an exact binomial statistical test. (**G**) Quantification of 5′ and 3′ terminal nucleotides of reads mapping to the upstream, antisense region of the 28S rDNA in I-PpoI transfected cells. The plots show the frequency of each base in drosha KO, *Dgcr8* KO and dicer KO mESCs, and in their respective parental lines (controls).

Since our experimental system allows us to reliably profile diRNAs using next-generation sequencing, we tested the proposed model that Drosha and Dicer mediate diRNA biogenesis (17,18,22). We first analysed diRNAs in drosha null-mutant mESCs (drosha KO line) to determine whether diRNA biogenesis requires Drosha. The drosha KO line, as expected, had severely reduced levels of Drosha-dependent miRNAs (Supplementary Figure S8). Surprisingly, however, the drosha KO cells were still able to produce diRNAs with the same directionality and overall distribution as the diRNAs produced in the parental cell line (Figure 5A). Furthermore, the loss of Drosha did not change the length of the diRNAs, and a diRNA population of length 21–22 nt was clearly visible in the drosha KO cells (Figure 5B and Supplementary Figure S9A). This observation was unexpected since it is well established that depletion of Drosha is detrimental to DNA repair (18,21,22,43).

The effect of Drosha on DSB repair has previously been investigated by knock-down (KD) experiments, not genetic KOs. To determine whether diRNA production is unaffected in the settings used in these previous studies, we knocked down Drosha in HeLa cells and analysed diRNA formation. Drosha KD affected neither diRNA production nor the distribution of read lengths (Supplementary Figure S10).

The microprocessor complex that processes primary miRNAs consists of Drosha bound to the DiGeorge Syndrome Critical Region 8 (DGCR8) protein. DGCR8 also acts independently of Drosha in the processing of small nucleolar RNAs (44) and in transcription-coupled nuclear excision repair following UV-induced DNA damage (45). We investigated whether DGCR8 plays a role in the biogenesis of diRNAs using a *Dgcr8* KO line, which is defective in miRNA biogenesis (Supplementary Figure S8). Similar to drosha KO cells, *Dgcr8* KO mESCs were able to generate diRNAs with a similar length distribution and sequence as those generated in control mESCs (Figure 5C and D). In summary, our results strongly suggest that neither Drosha nor DGCR8 are required for the biogenesis of diRNAs.

### Dicer is required for the biogenesis of a subset of diRNAs

We also analysed a dicer KO cell line to obtain further insight into the biogenesis of diRNAs. Dicer KO mESCs were deficient in canonical miRNAs (Supplementary Figure S8), as expected. Interestingly, I-PpoI expression in dicer KO mESCs still triggered diRNA production, as shown by small RNA-seq experiments (Figure 5E). Dicer processing is an essential step for diRNA production in plants, and it has been suggested that it is crucial also for diRNA biogenesis in mammalian cells (17,18,21,22,43). The ability of dicer KO mESCs to produce diRNAs was, therefore, intriguing. However, inspection of the size distribution of the diRNAs produced in the dicer KO line revealed the loss of the characteristic peak at 21–22 nt (Figure 5F and Supplementary Figure S9B).

The loss of the diRNAs of length 21–22 nt, but not of diRNAs of other lengths, in dicer KO mESCs further suggests the existence of at least two different populations of diRNAs. One of these populations is dependent on Dicer (diRNA-D) and is characterized by a length of 21–22 nt, whereas the Dicer independent population (diRNA-I) shows a broader length distribution.

Francia *et al.* reported that diRNAs produced at an I-SceI site in a reporter construct have a 5′ uracil and a 3′ guanine bias that is independent of the nucleotide composition of the sequences that surround the DSB (18). We asked whether the diRNA-D and diRNA-I populations derived from the endogenous I-PpoI cleavage site at the 28S locus also showed any 5′ or 3′ nucleotide preferences. We analysed the terminal residue of the 22-nt diRNAs, which we believe comprise a mix of diRNA-D and diRNA-I (the 22 nt fraction in Figure 5G), and we compared them with the sequences of reads of length 25 nt or longer, which we expected to be diRNA-Is (the 25 nt fraction in Figure 5G). The 22 nt fraction had a 5′ uracil bias that was not observed in the 25 nt fraction (Figure 5G, compare for instance line 5 with line 11). This bias was retained in both the drosha KO and *Dgcr8* KO cell lines (Figure 5G, line 2 and 4 compared to line 1 and 3). However, as expected, the 5′ uracil preference was not present in the 22 nt fraction of the dicer KO cells (Figure 5G, line 6 compared to line 5).

The 3′ end nucleotide features of the 22 and 25 nt fractions were also different. The 25 nt fraction, but not the 22 nt fraction, had a strong bias for a 3′ guanine (Figure 5G, compare for instance line 23 to line 17). Again, these 3′ end features were present also in drosha KO and *Dgcr8* KO cells (Figure 5G, lines 20 and 22 compared to lines 14 and 16, respectively), which further supports the conclusion that the microprocessor is not involved in diRNA biogenesis. Moreover, the remaining 22 nt diRNAs in the dicer KO cells had a very prominent 3′ guanine dominance that was otherwise characteristic of the 25 nt fraction (Figure 5G, line 18 compared to line 24), which agrees with the suggestion that most of the 21–22 nt diRNAs were Dicer products.

In summary, the analysis of terminal nucleotide biases confirmed the existence of two diRNA populations with different lengths and different end nucleotide signatures. DiRNA-Ds are 21–22 nt long and show a 5′ uracil bias that is typical of Dicer products (46,47), whereas diRNA-Is are of heterogeneous length and characterized by a pronounced guanine bias at the 3′ end.

### DiRNAs are loaded into Argonaute

**Figure 6.**
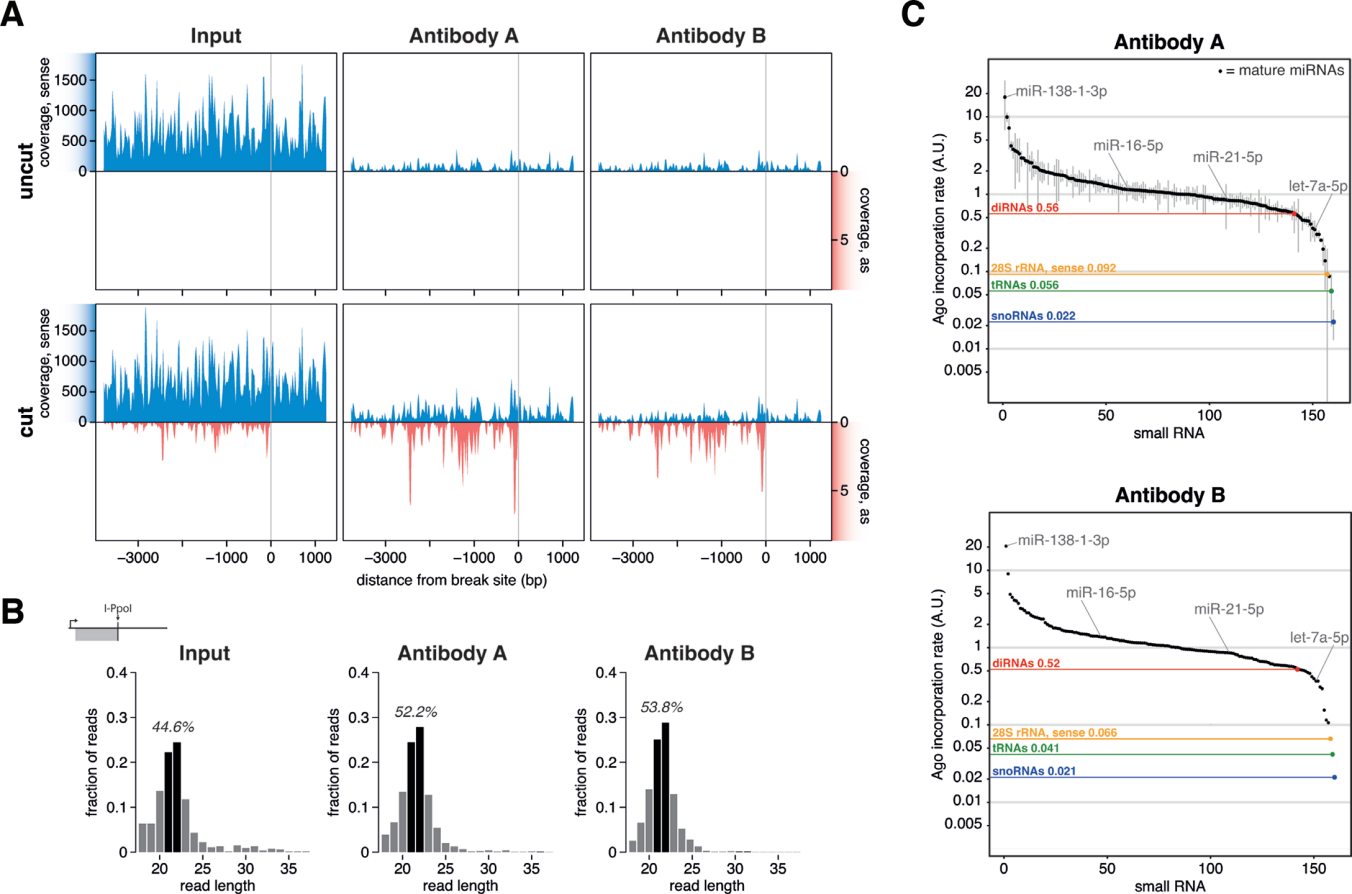
Endogenous diRNAs are incorporated into Argonaute. (**A**) Coverage of collapsed small RNA reads mapping to the 28S rDNA normalized to reads per million in Input and Argonaute RIP samples from either mock transfected (uncut) or I-PpoI expressing cells (cut). Coverage from three (antibody A) or two (antibody B) biological replicates were averaged. The grey vertical lines indicate the I-PpoI cleavage site. (**B**) Argonaute incorporation rates for miRNAs (black), snoRNAs (blue), tRNAs (green) or reads mapping to the antisense (diRNAs, red) or sense strand of the 28S rDNA (yellow). The Argonaute incorporation rate is estimated by fold-change (RIP/Input) normalized to the median miRNA fold-change. The plot shows averages from three (antibody A) or two (antibody B) biological replicates. Grey bars indicate the standard deviations. (**C**) Length distributions of diRNAs produced at the 28S rDNA (upstream, antisense) in Input or Argonaute RIP samples, as indicated in the figure. The percentage of the 21–22 nt population in each sample is displayed.

Dicer processed, functional small RNAs like miRNAs or siRNAs require incorporation into an Argonaute complex in order to be functional (48) and it has been proposed that diRNAs act to guide Argonaute proteins to DSBs, where the proteins can recruit DNA repair factors or DNA remodelling factors (21,22). However, direct evidence by sequencing of diRNA incorporation into Argonaute complexes in mammalian cells has so far been elusive. We performed RNA immunoprecipitation combined with small RNA sequencing (AGO RIP-seq) in HeLa cells transfected with I-PpoI to investigate whether diRNAs from the 28S locus were bound by Argonaute. We used two different antibodies against mammalian Argonautes, and we carried out negative control immunoprecipitations using an unrelated IgG antibody (IGG control). The specificity of the immunoprecipitation reactions was supported by quantification of input and immunoprecipitated RNAs and by electrophoretic analysis of the corresponding cDNA libraries (Supplementary Figure S11 and Table S6). As is commonly observed, the vast majority of reads retrieved from the AGO RIP-seq (∼90%) mapped to miRNAs (49). However, we could also readily detect diRNAs in AGO RIP samples. As expected, the levels of RIP-seq reads mapping to the forward strand of the ribosomal locus were very low compared to the input in samples with or without DSBs (Figure 6A, uncut and cut). In comparison to small RNA reads on the sense strand, reads mapping to the antisense strand were approximately ten times higher in the AGO RIP samples than in the input. The distribution of the AGO-bound diRNAs along the ribosomal locus and their length distribution were similar to those of diRNAs detected in the input (Figure 6A and B).

In order to estimate how efficiently diRNAs incorporate into AGO complexes, we defined the standard incorporation rate as the median fold change of miRNA levels in the AGO RIP and in the input. We found that diRNAs incorporated approximately at half the rate of a typical miRNA (Figure 6C), but their incorporation rate was significantly higher than that of tRNAs and snoRNAs (Figure 6C, green and blue). Interestingly, reads mapping to the sense strand of the 28S ribosomal locus were also incorporated at a higher rate than tRNAs or snoRNAs (Figure 6C, yellow), suggesting that at least some of these reads might not be background detection but reflect true diRNAs that cannot be identified in the input due to the very high background levels.

## DISCUSSION

We have used the I-PpoI endonuclease to produce sequence-specific DSBs in the human and mouse genomes and we provide evidence for the synthesis of dilncRNAs at both unique and repetitive genomic loci in mammalian cells. Moreover, we have carried out extensive small RNA sequencing and we show that the rDNA locus produces significant levels of small diRNAs, whereas unique genic and intergenic loci do not. A small RNA response to DNA damage in the rDNA was already revealed by pioneering studies in *Neurospora* (12,28). There could be several reasons why diRNAs are robustly detected at the ribosomal locus but not at unique loci. First, there are multiple copies of the rDNA cassette in the human genome (50), which results in a considerable amplification of transcript levels in the RNA-seq experiments. However, we show that the copy number is not sufficient to explain the difference observed between the repetitive rDNA and unique loci. On the other hand, diRNAs have been linked to DNA repair by HR (21) and, as such, the circumstance that DSBs in the rDNA are predominantly repaired by HR might favour diRNA detection (3). Furthermore, it has been proposed that diRNA production depends on the base pairing of the pre-diRNA synthesized *de novo* in sense with transcripts that were made at the host locus before the DSB arose (13,29). The high transcription rates of rRNA may therefore be ideal for diRNA formation. Interestingly, diRNA-like small RNAs termed telomeric DNA damage response RNAs have been described at deprotected telomeres, which are also highly repetitive, transcribed genomic regions (24). We therefore propose that it is the combination of transcription and the repetitive nature of these loci that triggers the formation of diRNAs.

Our study confirms previous observation on the generation of dilncRNAs from DSBs (13,16,17), but we did not find evidence for the synthesis of dilncRNAs extending towards the DSBs. The bidirectional *de novo* transcription proposed by Michelini *et al.* (16) would result in the generation of diRNAs mapping both downstream and upstream of the DSB. However, the diRNAs in the 28S locus show a very distinct distribution and map exclusively upstream of the I-PpoI cleavage site, which supports the idea that diRNA biogenesis relies on pre-existing sense transcripts, as previously proposed by others (13,23).

**Figure 7.**
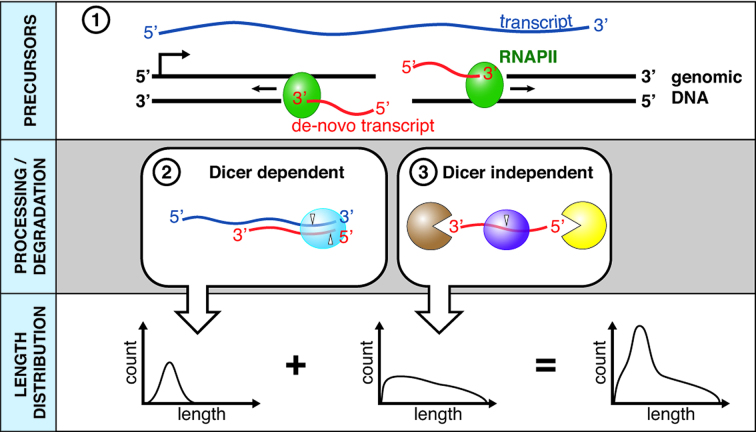
Model for the biogenesis of Dicer-dependent and Dicer-independent diRNAs at endogenous DSBs in the mammalian genome. See main text for details.

We have revealed the existence of two different diRNA populations that result from the processing of dilncRNAs at repetitive, transcribed loci and we propose a model for their biogenesis. According to this model, the free DNA ends at the DSB recruit RNAPII, which results in the synthesis of dilncRNAs (Figure 7, box 1). We have been able to detect dilncRNAs extending from DSBs in both the 28S rDNA locus and the *Ryr2* gene. The newly made dilncRNAs are able to anneal with already existing transcripts made at the same locus, and double-stranded RNAs are produced. These double-stranded RNAs can be processed by Dicer into diRNAs with the characteristic length of 21–22 nt (Figure 7, box 2).

DNA–RNA hybrids form at DSBs, as shown by others (15,51), which implies that the dilncRNAs also anneal with the template DNA. Moreover, our model proposes that a fraction of the dilncRNAs are degraded, probably by different endoribonucleases and exoribonucleases, which generates the population of diRNA-Is with a broad range of lengths that we observe in Dicer deficient cells (Figure 7, box 3). Thus the diRNA length profile of wild-type cells is a combination of the profiles of diRNA-Ds and diRNA-Is.

We show that Drosha and DGCR8 are not necessary for diRNA biogenesis. This observation is surprising because Drosha is recruited to DSBs, Drosha depletion results in severe HR defects (18,21,22,43), and DGCR8 is involved in the repair of UV-induced DNA damage (45). Our results imply that the roles of these proteins in DNA repair are independent of diRNA biogenesis. In agreement with this finding, a recent report proposed a role for Drosha in DNA end resection and in resolving DNA:RNA hybrids at DSBs (14).

We did not find evidence for small diRNA production at unique genomic sites. Due to the lack of precise timing control in the I-PpoI transfection system, we cannot formally rule out the possibility that diRNAs are synthesized and degraded very rapidly after DSB induction. However, similar conclusions were reached in a very recent study based on the use of DIvA cells, which allow a tighter time control (14). Therefore, we conclude that the diRNA response observed at repetitive genomic sites is not a universal landmark of DSBs.

The model that we present here assumes that RNAPII is able to engage in transcription at DSBs, but the mechanism by which the transcription machinery is recruited to the damaged DNA is unknown. The dilncRNAs synthesized upon I-PpoI cleavage in the 28S rDNA are the product of RNAPII although this polymerase is not particularly enriched in the nucleolus. *De novo* transcription by RNAPII is a general phenomenon that occurs also at DSBs in unique genomic sequences, as shown by us and others (15,16). Studies from Förstemann and coworkers showed that transcription at free DNA ends can take place in the absence of a promoter in insect cells (13) and research in the chromatin field has revealed that specialized regulatory complexes, such as FACT, are necessary to suppress inappropriate transcription initiation from within coding regions (52). These observations support the view that transcription initiation by RNAPII in the absence of a promoter is not uncommon in eukaryotic cells and could occur at DSBs. Whether any of the early DDR events plays an active role in the recruitment of RNAPII to DSBs remains to be investigated.

Our RNA-seq data allows us to draw robust and quantitative conclusions on the abundance of diRNAs in mammalian cells. We have estimated that approximately 30 diRNAs per cell are derived from the 28S loci upon I-PpoI cleavage, including both diRNA-Ds and diRNA-Is, and that their rate of incorporation into Argonaute complexes is 0.5. We do not know whether these two diRNA populations have similar, or any, functions in DSB repair, but their loading into Argonaute complexes would allow them to act as guides for the recruitment of histone modifiers and DNA repair factors, as proposed in previous studies (21,22). Similar estimates for each of the eight unique I-PpoI targets in the human genome, assuming that the few small RNAs mapping to these loci are diRNAs, result in abundances that are far below one Argonaute-loaded diRNA per DSB.

Our estimates of diRNA abundance are based on experimental determinations of miR-15a abundance and I-PpoI cleavage, but rely also on a number of assumptions. For example, we have presumed that the miR-15a abundances determined in previous studies (35,37) are applicable to the HeLa cells we have used for the present study although we cannot rule out minor variations that would influence the accuracy of our estimates. We cannot exclude either the possibility that higher levels of unstable diRNAs are produced at earlier time points. Furthermore, we have use collapsed small RNA reads to exclude PCR amplification artifacts, which might result in an underestimation of diRNA levels. In spite of these limitations, the depth of our small RNA-seq analysis and the total absence of reads at many of the analysed DSBs support the conclusion that diRNAs from unique genomic loci are not produced or produced at exceedingly low levels, which raises doubts about their functional significance.

## DATA AVAILABILITY

Raw sequence data in the form of fastq files have been deposited in the Gene Expression Omnibus GEO under accession number GSE106764.

## Supplementary Material

Supplementary Data
