# Supplementary material for: Next-generation sequencing reveals two populations of damage-induced small RNAs at endogenous DNA double-strand breaks
Source: Nucleic Acids Res. 2018 Nov 10;46(22):11869–82. doi: 10.1093/nar/gky1107 (PMC6294500; doi:10.1093/nar/gky1107)
Supplement: Supplementary Data [file gky1107_supplemental_files.pdf]

Supplementary Figure 1

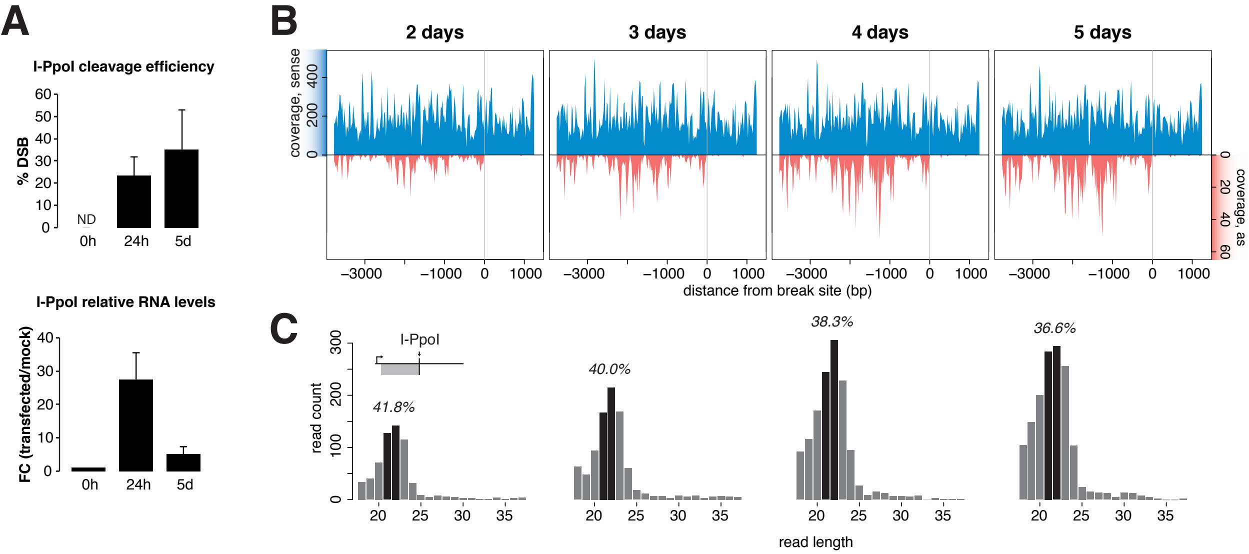

Supplementary Figure 1. DiRNAs can be detected for at least 5 days after I-PpoI transfection. (A) Percentage of DSBs in the Ryr2 locus (upper panel) and I-PpoI expression levels (lower panel) after transfection of HeLa cells with I-PpoI and cultivation for the indicated times. The percentage of DSBs was quantified by qPCR on genomic DNA with primers spanning the I-PpoI recognition motif in I-PpoI transfected cells and was normalized to ARPP, which is not cleaved by I-PpoI. I-PpoI expression levels were determined by RT-qPCR. All measurements were performed in three independent experiments. The histograms show average values and the error bars represent standard deviations. (B) Small RNA reads of HeLa cells transfected with I-PpoI and incubated for the indicated times. The reads were mapped to the 28S rDNA locus and normalized to total collapsed 28S rRNA read counts per million. The grey vertical lines indicate the I-PpoI cleavage site. (C) Read length distribution of collapsed reads mapping to the upstream, antisense region of the 28S rDNA locus in a time course after I-PpoI transfection. The black bars indicate read lengths of 21-22 nt. The percentage of the 21-22 nt fraction is indicated in the figure.

Supplementary Figure 2

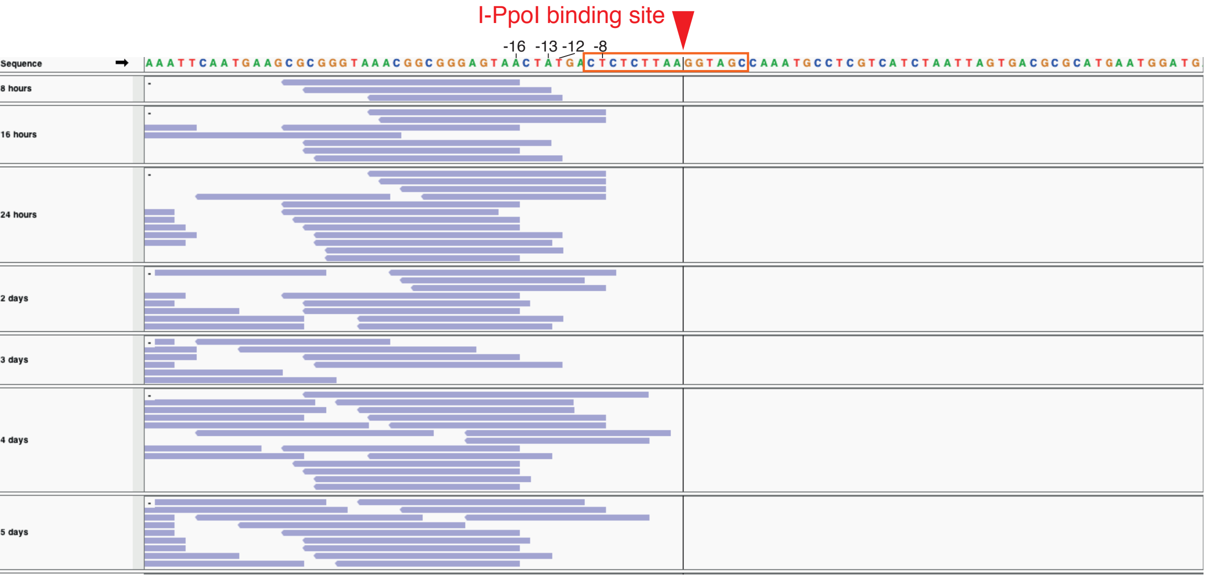

Supplementary Figure 2. Mapping of diRNAs close to the I-PpoI cleavage site. The plot shows collapsed small RNA reads that map to the 28S rDNA antisense locus of HeLa cells transfected with I-PpoI. The red arrowhead points to the I-PpoI site on the sense strand and the most prominent starting positions for the reads in respect to the DSB are highlighted by numbers on top of the plot.

Supplementary Figure 3

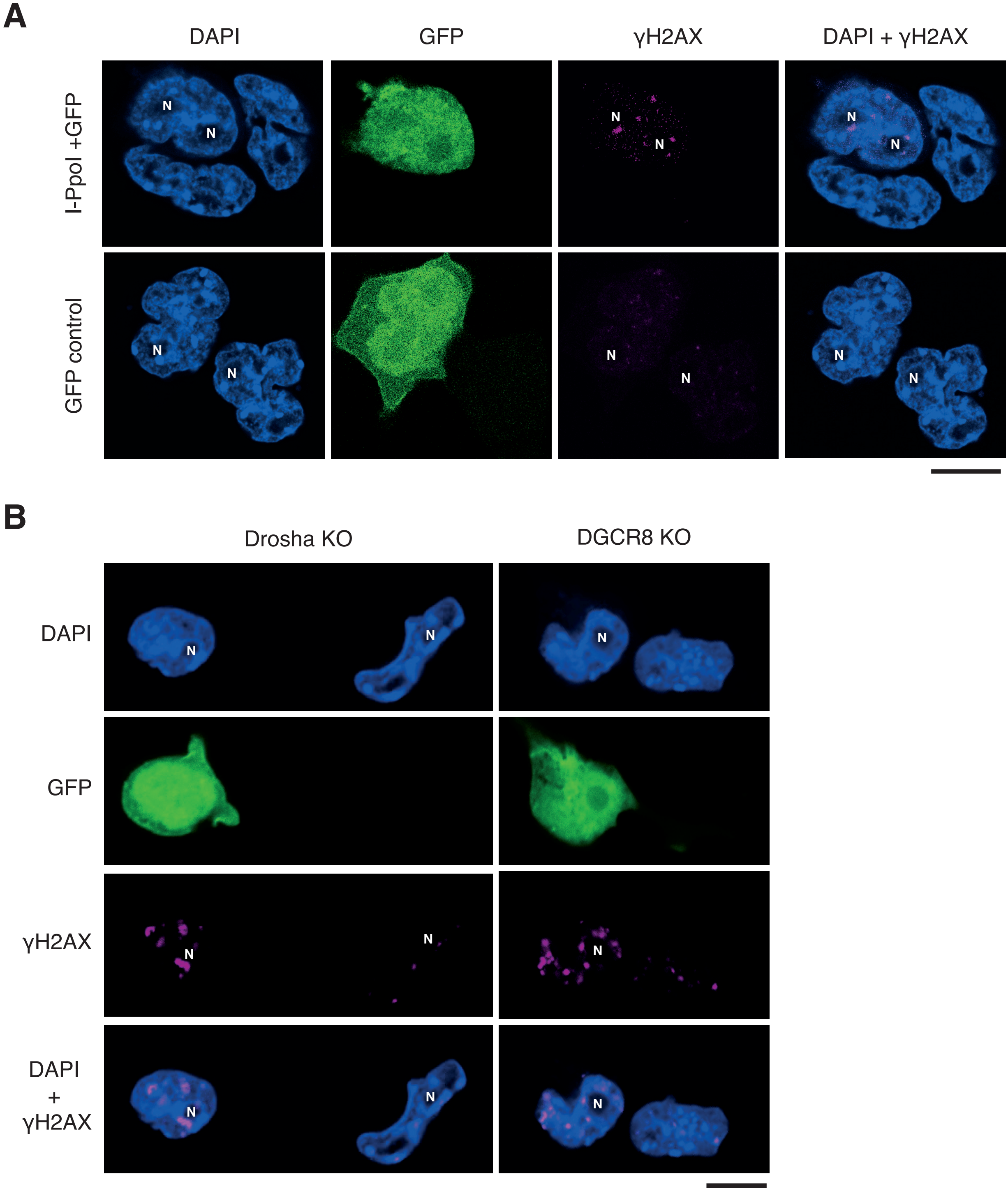

Supplementary Figure 3. I-PpoI induces DSBs in mESCs. (A) Mouse ESCs were transfected with either a mixture of I-PpoI and GFP expression plasmids (top row) or with GFP plasmid alone (bottom row). Subsequently, the cells were cultured for 24 hours and stained for γH2AX to identify DSBs. DAPI was used to visualize the nucleus and identify the nucleoli (N). (B) Drosha KO and Dgcr8 KO mESCs were transfected with a mixture of I-PpoI and GFP expression plasmids and proessed for immunofluorescence as above. All images are confocal sections taken at the same magnification. The scale bars represent 10 μm.

Supplementary Figure 4

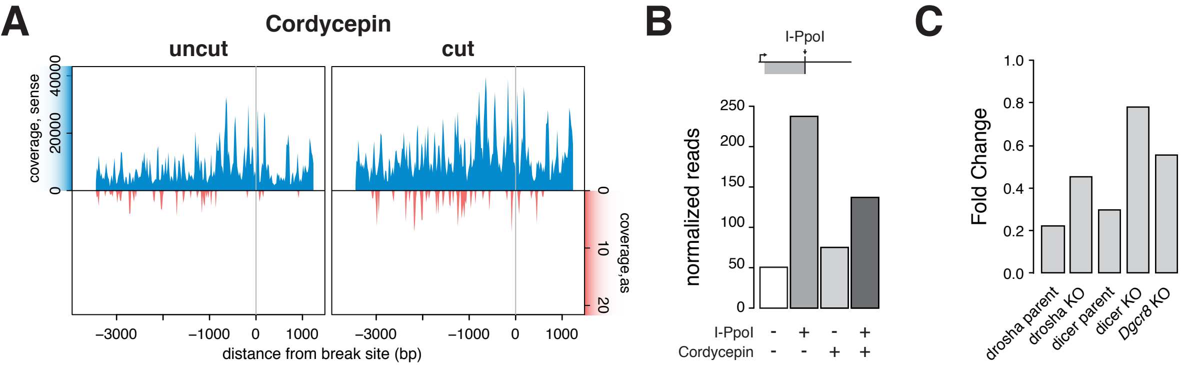

Supplementary Figure 4. DiRNA production in repetitive regions requires transcription after DSB induction. (A) Coverage plot of small RNAs extracted from mESCs mock transfected (uncut) or I-PpoI transfected (cut) and cultured for 36 hours. Cells were treated with Cordycepin for 12 hours prior to harvest. Plots are normalized to spike-ins. (B) Quantification of collapsed reads, expressed as reads per million spike-ins, mapping to the upstream antisense region of the 28S rDNA locus in the experiments shown in (A) and Figure 2A. (C) The effect of cordycepin on RNAPII transcription was assessed by measuring the Fold change of intronic versus exonic GAPDH (pre)mRNA levels in untreated mESCs and mESCs treated with Cordycepin for 12 hours. The cells were harvested 24 hours after transfection with I-PpoI. Each bar represents the average of two independent replicates. The reduced intronic versus exonic ratio shows decreased transcriptional activity.

Supplementary Figure 5

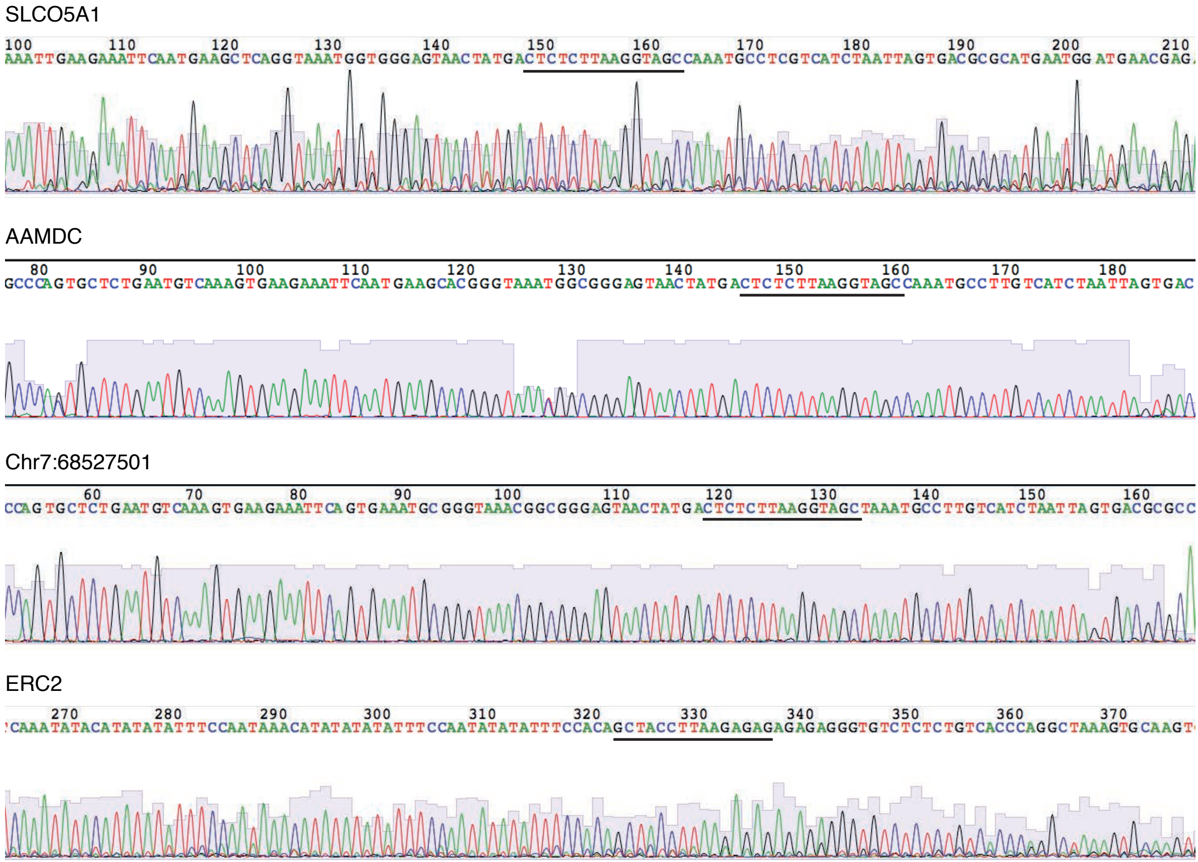

Supplementary Figure 5. Sanger sequencing of PCR products amplified from the same HeLa cells used in this study. The PCR products correspond to four selected unique I-PpoI sites in the human genome. The I-PpoI target sequences are underlined in the plots. The four I-PpoI target sequences are intact.

Supplementary Figure 6

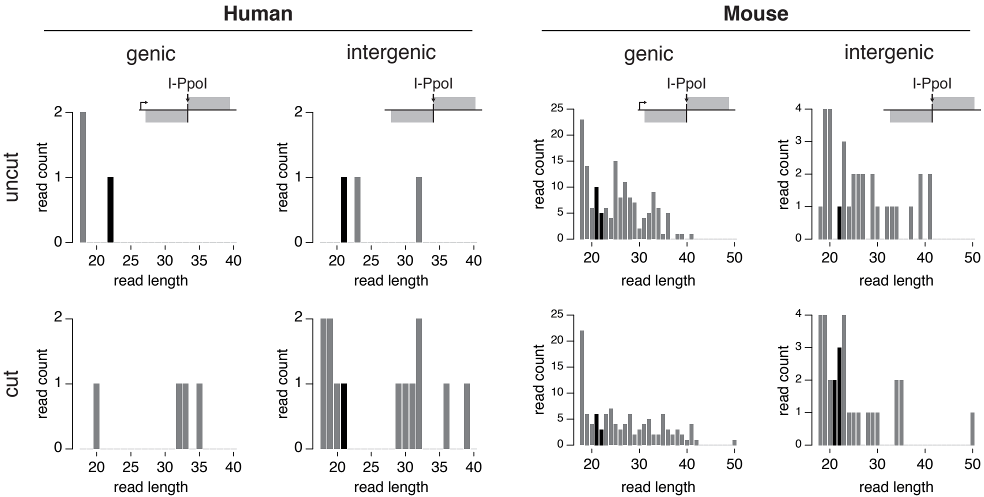

Supplementary Figure 6. Read length distribution of small RNA reads mapping to unique genomic loci harboring an I-PpoI consensus sequence. The plots show collapsed, uniquely mapping reads mapping within a region of 5 kb upstream or downstream of the I-PpoI sites in mock transfected cells (uncut) and in cells expressing I-PpoI (cut). Genic and intergenic are as defined in Figure 3A. The analysis includes the I-PpoI sites that are listed in the Supplementary Tables 2 and 3.

Supplementary Figure 7

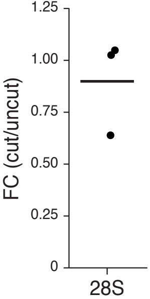

Supplementary Figure 7. The increase in RNA levels observed at the 28S rDNA locus (upstream and antisense to I-PpoI site) in cells that express I-PpoI is not due to a general effect of DDR on nucleolar activity. DSBs were induced in DIvA cells by 4-OHT and the expression of antisense RNA upstream of the 28S rDNA locus was determined by ss-RT-qPCR and normalized to GAPDH. The plot shows the fold change between induced (cut) and uninduced (uncut cells) in three independent experiments. The experiment shows that DSBs produced in different genomic loci do not induce RNA synthesis at the 28S locus. The p-value of a two-tailed one-sample test is 0.655.

Supplementary Figure 8

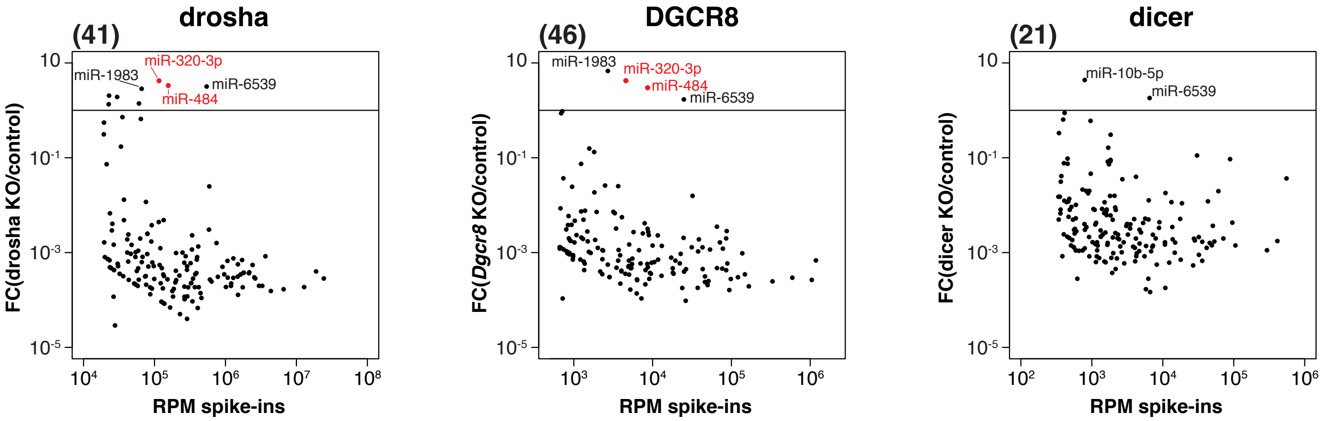

Supplementary Figure 8. MiRNA abundance is strongly reduced in drosha, *Dgcr8* and dicer KO mESC lines. Fold change of miRNA levels in the indicated mESC KO lines compared to control cell lines. The reads were mapped against all miRNA hairpins and the read counts are expressed as reads per million spike-ins. The 200 most abundant miRNAs in the control lines were used for the subsequent analysis. The figures in parenthesis in the top left corner of each plot indicated the number if miRNAs listed in the top 200 that were undetectable in the KO lines. MiRNAs labeled in red were shown previously to be independent of Drosha and DGCR8 processing.

Supplementary Figure 9

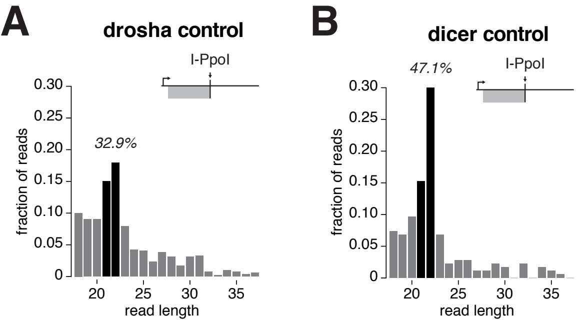

Supplementary Figure 9. Read length distribution of diRNAs in the parental control mESC lines. (A,B) Small RNA analysis of the parental cell lines that were used for the drosha (A) and dicer (B) KO experiments shown in Figure 5. The plots show average read length distributions of diRNAs (28S rDNA, upstream, antisense) in cells transfected with the I-PpoI plasmid from three (drosha) or two (dicer) biological replicates.

Supplementary Figure 10

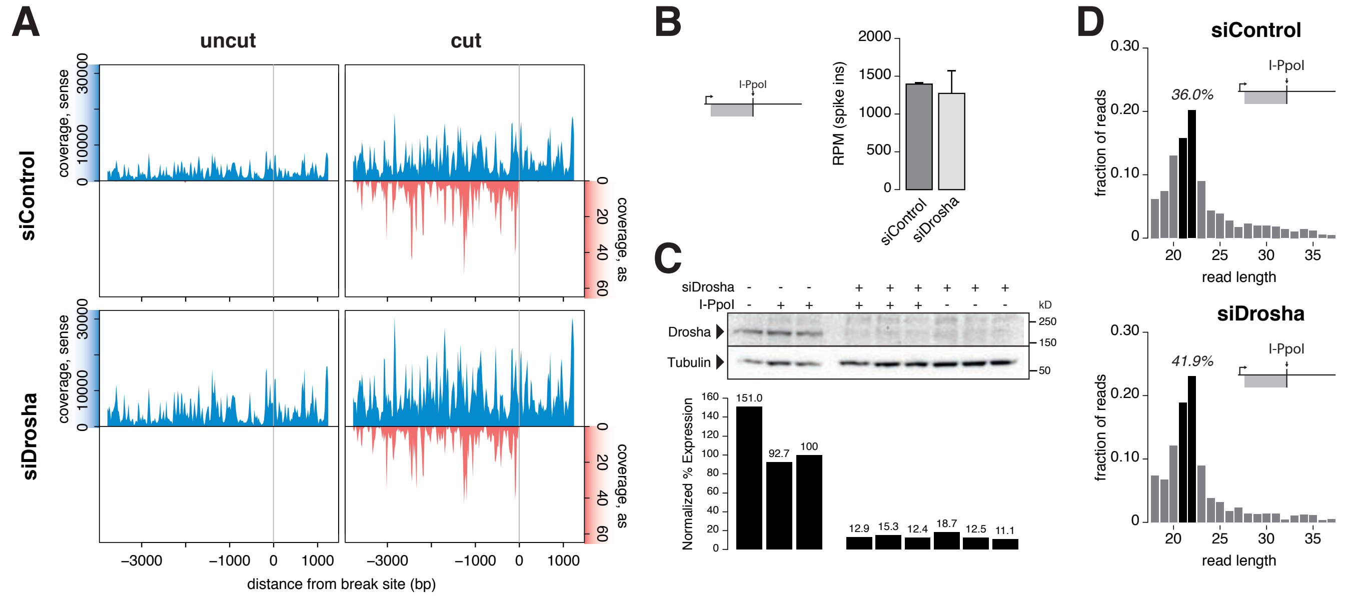

Supplementary Figure 10. DiRNA production is unperturbed by Drosha depletion in HeLa cells. (A) The plots show small RNA reads mapping to the 28S rDNA locus in HeLa cells either transfected with control siRNA (siControl) or siRNA targeting Drosha (siDrosha). To produce a DSB, the "cut" samples were transfected with the I-PpoI expression plasmid. The plots show the average coverage of three replicates expressed as coverage per million spike-ins. The grey vertical lines indicate the position of the I-PpoI cleavage site. (B) Quantification of reads mapping to the upstream, antisense region of the 28S rDNA locus shown in (A), expressed as reads per million spike-ins. The bars represent averages of the three independent experiments and the error bars represent standard deviations. (C) Western blot analysis of Drosha in the samples used for small RNA sequencing in (A). Tubulin was used as a loading control for normalization. The mobility of molecular mass standards is shown to the right. The relative Drosha levels were quantified and are shown in the plot below the blot. (D) Read length distribution of collapsed reads mapping to the upstream, antisense strand region of the 28S rDNA locus in I-PpoI transfected HeLa cells, either control (top) or Drosha-depleted (bottom). The black bars indicated reads of 21 and 22 nt. The percentage of the 21-22 nt fraction is indicated.

Supplementary Figure 11

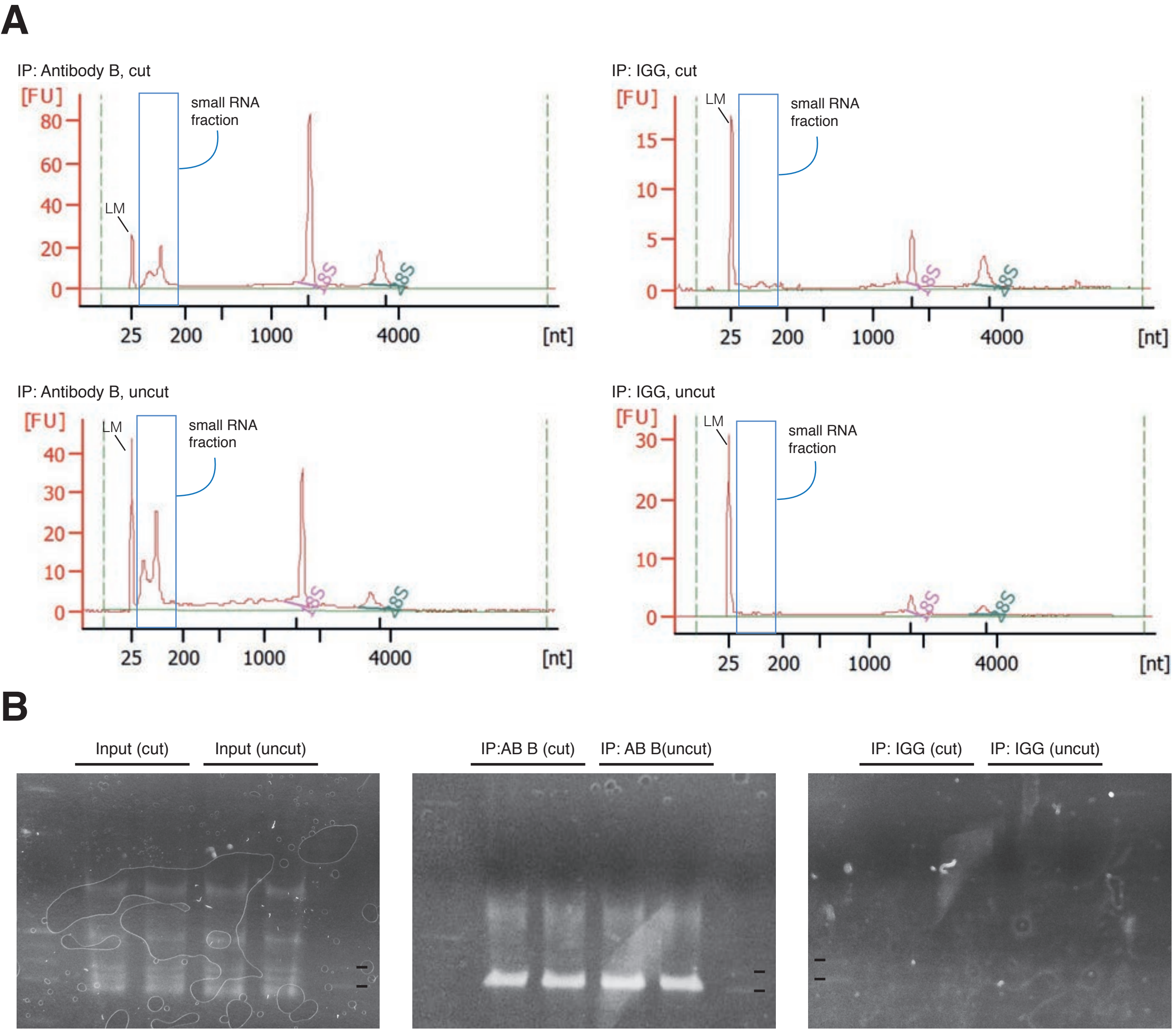

Supplementary Figure 11. Specificity of the Argonaute immunoprecipitation reactions. (A) Example of bioanalyzer traces for RNAs purified after Argonaute IP using antibody B in a cut or uncut sample (replicate II) and the corresponding negative control (IgG IP). The peak of the lower marker (LM) and the small RNA fraction are highlighted. (B) Polyacrylamide gels with the cDNA libraries of the samples shown in (A) and the corresponding input samples after Illumina library preparation. Each library was loaded in two lines. Libraries were size selected by gel purification of the cDNA situated between the two lines as indicated. The RNA and cDNA concentration for each sample can be found in the Supplementary Table 5. The low amounts of RNA detected in the IgG IP samples supports the specificity of the IP experiments.

Sample Name	Cell type	Treatment(s)	Reads after QC
4_siDro_cut	HeLa	I-PpoI transfection	8317775
5_siDro_cut	HeLa	I-PpoI transfection	12109309
6_siDro_cut	HeLa	I-PpoI transfection	16650483
16_siCont_cut	HeLa	I-PpoI transfection, control siRNA transfection	10439953
17_siCont_cut	HeLa	I-PpoI transfection, control siRNA transfection	9671264
18_siCont_cut	HeLa	I-PpoI transfection, control siRNA transfection	8940643
28_siCont_cut_Cord	HeLa	I-PpoI transfection, control siRNA transfection, Cordycepin	7365731
29_siCont_cut_Cord	HeLa	I-PpoI transfection, control siRNA transfection, Cordycepin	4610288
30_siCont_cut_Cord	HeLa	I-PpoI transfection, control siRNA transfection, Cordycepin	9388639
JP4_4h_BC13_AGTCAA	HeLa	I-PpoI transfection	12703545
JP5_8h_BC14_AGTTCC	HeLa	I-PpoI transfection	21537808
JP6_16h_BC15_ATGTCA	HeLa	I-PpoI transfection	10731850
JP7_24h_BC16_CCGTCC	HeLa	I-PpoI transfection	25711049
JP9_48h_BC17_GTAGAG	HeLa	I-PpoI transfection	13852496
JP10_3d_BC18_GTCCGC	HeLa	I-PpoI transfection	12856067
JP11_4d_BC19_GTGAAA	HeLa	I-PpoI transfection	16059397
JP12_5d_BC20_GTGGCC	HeLa	I-PpoI transfection	15361275
1_siDro_unc	HeLa	Mock transfection, siDrosha transfection	6323292
2_siDro_unc	HeLa	Mock transfection, siDrosha transfection	5407436
3_siDro_unc	HeLa	Mock transfection, siDrosha transfection	7845335
13_siCont_unc	HeLa	Mock transfection, control siRNA transfection	9747729
14_siCont_unc	HeLa	Mock transfection, control siRNA transfection	11721556
15_siCont_unc	HeLa	Mock transfection, control siRNA transfection	7394595
25_siCont_unc_Cord	HeLa	Mock transfection, control siRNA transfection	9941093
26_siCont_unc_Cord	HeLa	Mock transfection, control siRNA transfection	3422307
27_siCont_unc_Cord	HeLa	Mock transfection, control siRNA transfection	5455818
JP1_nonT_BC10_TAGCTT	HeLa	-	4327701
JP2_M0h_BC11_GGCTAC	HeLa	Mock transfection	13751472
JP3_M24h_BC12_CTTGTA	HeLa	Mock transfection	17201147
4_mESC_cut	mESC, parental Drosha KO	I-PpoI transfection	6845360
5_mESC_cut	mESC, parental Drosha KO	I-PpoI transfection	5601322
10_cont_cut	mESC, parental Drosha KO	I-PpoI transfection	26247920
11_cont_cut	mESC, parental Drosha KO	I-PpoI transfection	17113908
12_cont_cut	mESC, parental Drosha KO	I-PpoI transfection	31233489
16_DGCR8_cut	mESC, DGCR8 KO	I-PpoI transfection	5324524
18_DGCR8_cut	mESC, DGCR8 KO	I-PpoI transfection	1032695
17_DroKO_cut	mESC, Drosha KO	I-PpoI transfection	6228039
16_DroKO_cut	mESC, Drosha KO	I-PpoI transfection	27738642
18_DroKO_cut	mESC, Drosha KO	I-PpoI transfection	31989552
22_DcrKO_cut	mESC, Dicer KO	I-PpoI transfection	2612257
23_DcrKO_cut	mESC, Dicer KO	I-PpoI transfection	3800299
34_mECS_cut_cord	mESC, parental Drosha KO	I-PpoI transfection, Cordycepin	6128908
35_mECS_cut_cord	mESC, parental Drosha KO	I-PpoI transfection, Cordycepin	5631757
C_DcrPar_cut	mESC, parental Dicer KO	I-PpoI transfection	8674386

D_DcrPar_cut	mESC, parental Dicer KO	I-PpoI transfection	5165356
1_mESC_unc	mESC, parental Drosha KO	Mock transfection	3432390
2_mESC_unc	mESC, parental Drosha KO	Mock transfection	6486411
7_cont_unc	mESC, parental Drosha KO	Mock transfection	30423455
8_cont_unc	mESC, parental Drosha KO	Mock transfection	13085968
9_cont_unc	mESC, parental Drosha KO	Mock transfection	30391510
13_DGCR8_unc	mESC, DGCR8 KO	Mock transfection	4759247
14_DGCR8_unc	mESC, DGCR8 KO	Mock transfection	6125303
13_DroKO_unc	mESC, Drosha KO	Mock transfection	25660139
14_DroKO_unc	mESC, Drosha KO	Mock transfection	22815180
15_DroKO_unc	mESC, Drosha KO	Mock transfection	6477722
19_DcrKO_unc	mESC, Dicer KO	Mock transfection	2398187
20_DcrKO_unc	mESC, Dicer KO	Mock transfection	3142807
31_mECS_unc_cord	mESC, parental Drosha KO	Mock transfection, Cordycepin treatment	6177500
32_mECS_unc_cord	mESC, parental Drosha KO	Mock transfection, Cordycepin treatment	2596212
A_DcrPar_unc	mESC, parental Dicer KO	Mock transfection	4371200
B_DcrPar_unc	mESC, parental Dicer KO	Mock transfection	4535307

Supplementary Table 1
Small RNA-seq libraries used for Figure 3, Supplementary Figure 6 and Supplementary Table 4.

Chromosome	I-PpoI site	Gene	Gene strand
1	46076099	Dnah7b	fw
1	113904189	-	-
1	191121007	-	-
3	5860455	-	-
3	121249941	tmem56	re
4	72028087	-	-
5	114852064	-	-
7	120754166	vw3a	fw
9	113566737	AU023762	re
12	80441534	-	-
13	45898843	Atxn1	re
14	56694153	Mphosph8	fw
17	6941939	Rsph3b	re
17	7952365	rsph3a	fw
17	24666018	-	-
X	116287269	-	-

Supplementary Table 2
I-PpoI recognition sequences in the mouse genome (mm10).

Chromosome	I-PpoI site	gene	gene strand
1	58453288	DAB1	Re
1	237766424	RYR2	Fw
2	133037436	-	-
3	56364072	ERC2	Re
7	68527501	-	
8	70602504	SLCO5A1	Re
11	77597591	AAMDC/INTS4	Fw/Re
X	108297464	-	-

Supplementary Table 3
I-PpoI recognition sequences in the human genome (hg19).

Locus	sites/cell	%DSB (%cleaved)	nr. of reads (uncut)	nr. of reads (cut)	nr. of diRNAs (cut)/cell	nr. of diRNAs (cut)/cell/cleaved site
28S	75‡	32.4‡	29	8835	28.6	1.12
Ryr2	2	56.6‡	0	0	0	0
Dab1	2	36.5‡	0	0	0	0
SLCO5A1	2	51.3‡	0	0	0	0
AAMDC	2	0‡	3	4	0.013	0.03*
ERC2	2	nd	0	0	0	0*
Chr2:133037436	2	nd	1	6	0.019	0.05*
Chr7:68527501	2	37.4‡	0	0	0	0
ChrX:108297464	2	nd	2	7	0.023	0.06*

Supplementary Table 4
Estimation of diRNA copies/cell in HeLa cells
The table shows the number of un-normalized collapsed reads that map to a region of 10 kb which extends 5 kb upstream and downstream of the I-PpoI site and the estimated number of diRNAs per cell at the indicated loci. The number of diRNAs per cell was estimated by normalization to known miRNA abundances in each sample (see Materials and Methods for details). The number of diRNAs per cleaved site are the number of diRNAs per cell divided by the number of sites per cell and corrected by the cleavage rate. The samples used are listed in the Supplementary Table 1 (HeLa, cut samples only).
nd: not determined
‡ experimentally determined by qPCR
* 20% DSB was assumed for calculations of nr. diRNAs/copy at loci with no DSBs or unknown %DSB

Locus	nr. of reads (uncut)	nr. of reads (cut)
28S	513	2486
DNAH7b	1	1
2310007B03RiK	64	41
TMEM56	1	1
VW3a	0	1
AU023762	2	2
ATXN1	0	0
MPHOSPH8	80	62
RSPh3b	3	3
RSPH3a	1	1
Chr1:113904189	2	1
Chr1:191121007	0	0
Chr3:5860455	0	0
Chr3:138077304	0	0
Chr4:72018087	1	1
Chr5:114852064	2	2
Chr10:55572700	0	0
Chr12:80441534	1	4
Chr17:24666018	25	22
ChrX:116287269	0	0

Supplementary Table 5
Distribution of reads in unique loci of mESCs
The samples used are listed in Supplementary Table 1 (mESC). The table shows the number of un-normalized, collapsed reads mapping to a region of 10kb that extends 5kb upstream and downstream to the I-PpoI sites.

		RNA return after RIP /input small RNAseq			cDNA library, total yield		
		**I**	**II**	**III**	**I**	**II**	**III**
**Input**	**cut**	1000 ng	1000 ng	1000 ng	12 ng	16 ng	9.8 ng
	**uncut**	1000 ng	1000 ng	1000 ng	6.1 ng	17 ng	11 ng
**Antibody A**	**cut**	15 ng	37 ng	11 ng	53 ng	67 ng	53 ng
	**uncut**	41 ng	17 ng	13 ng	53 ng	54 ng	50 ng
**Antibody B**	**cut**	-	28 ng	7 ng	-	60 ng	40 ng
	**uncut**	-	25 ng	5 ng	-	71 ng	34 ng
**IGG**	**cut**	3 ng	3 ng	7 ng	0.8 ng	1.1 ng	1.2 ng
	**uncut**	5 ng	4 ng	2 ng	0.9 ng	1.3 ng	0.6 ng

Supplementary Table 6
Amount of RNA returned by Ago-RIP subsequently used for small RNA library preparation and total yields returned by library preparation.
